# Impact of online patient reminders to improve asthma care: A randomized controlled trial

**DOI:** 10.1371/journal.pone.0170447

**Published:** 2017-02-03

**Authors:** Andrew C. Pool, Jennifer L. Kraschnewski, Jennifer M. Poger, Joshua Smyth, Heather L. Stuckey, Timothy J. Craig, Erik B. Lehman, Chengwu Yang, Christopher N. Sciamanna

**Affiliations:** 1 Department of Public Health, Temple University College of Health Professions and Social Work Philadelphia, Pennsylvania, United States of America; 2 Center for Obesity Research and Education (CORE), Temple University School of Medicine, Philadelphia, Pennsylvania, United States of America; 3 Department of Medicine, Penn State College of Medicine, Hershey, Pennsylvania, United States of America; 4 Department of Pediatrics, Penn State College of Medicine, Hershey, Pennsylvania, United States of America; 5 Department of Public Health Sciences, Penn State College of Medicine, Hershey, Pennsylvania, United States of America; 6 Department of Biobehavioral Health, Pennsylvania State University, State College, Pennsylvania, United States of America; Public Library of Science, FRANCE

## Abstract

**Importance:**

Asthma is one of the most burdensome chronic illnesses in the US. Despite widespread dissemination of evidence-based guidelines, more than half of the adults with asthma have uncontrolled symptoms.

**Objective:**

To examine the efficacy of an online tool designed to improve asthma control.

**Design:**

12-month single blind randomized controlled trial of the online tool (Intervention condition, IC) versus an active control tool (CC).

**Setting:**

Patients enrolled in an insurance plan.

**Participants:**

Participants were 408 adults (21–60 years of age) with persistent asthma.

**Intervention:**

At least once each month and before provider visits, participants in the IC answered questions online about their asthma symptoms, asthma medications and asthma care received from providers, such as an asthma management plan. The tool then provided tailored feedback to remind patients 1) to ask health care providers specific questions that may improve asthma control (e.g., additional controller medications) and 2) to consistently perform specific self-care behaviors (e.g., proper inhaler technique). Participants in the CC received similar questions and feedback, yet focused instead on preventive services unrelated to asthma control (e.g., cancer screening).

**Main outcome measures:**

The main outcome measure was asthma control, as assessed by the 5-question Asthma Control Test (ACT). Secondary outcomes included quality of life, medication use and healthcare utilization (e.g., emergency department visits).

**Results:**

After 12 months, 323 participants completed follow-up measures (79.2%). Participants in the IC reported a greater mean improvement in the ACT score than participants in the CC (2.3 vs. 1.2; p = 0.02) and 9 of 11 individual asthma control survey items showed non-significant improvements favoring the IC. No differences were observed in medication adherence, number of asthma controller medications or health care utilization.

**Conclusion and relevance:**

Simple and brief online patient reminders improved asthma control among insured patients. Although future studies are needed to understand the mechanism of the improvement, the magnitude of the effect on asthma control was similar to the addition of an additional controller medication. Given the widespread use of the Internet, simple tools such as this may be useful for improving the control of other chronic diseases as well.

**Trial registration:**

This study is registered at clinicaltrials.gov, NCT00921401, “Improving the Quality of Asthma Care Using the Internet”

## Introduction

Asthma is one of the most burdensome chronic illnesses, affecting approximately 25 million people in the US and accounting for nearly 500,000 hospitalizations, 1.9 million emergency department visits and direct medical costs of $18 billion annually [1–3]. Despite widespread dissemination of evidence-based guidelines and effective treatment that have existed for decades, most patients with asthma still have uncontrolled symptoms [4–6]. According to the National Asthma Education and Prevention Program (NAEPP) guidelines, poor asthma control is defined as having daytime symptoms or requiring a short-acting inhaler more than twice weekly, awakening from sleep due to asthma symptoms, or having to reduce activities due to asthma [7]. In a random sample of adults with health insurance, 51.7% had uncontrolled asthma based on an Asthma Control Test (ACT) score of <20 [4]. Similar observations of poor asthma control have been reported in asthma management program evaluations [5,6]. Interventions aimed at improving self-management of asthma control are crucial to reducing the adverse physical and economic impacts associated with this disease.

Though patient non-adherence to controller medicines is a contributing factor to poor asthma control [8], there is ample evidence to suggest that patients are also not prescribed enough controller medications and do not receive proper counseling and instruction (i.e. proper use of an inhaler) to help self-manage their asthma. It is estimated, for example, that only 55% of adults with asthma are taught to recognize early symptoms of asthma, only 47% are instructed to change their environment to improve asthma control, and only 33% have ever received an asthma management plan [9,10]. Slejko and colleagues studied a representative sample of 102,544 US adults with asthma and observed that only 60% of those using three or more canisters of a quick-relief inhaler in the past 90 days also used a long-term controller medication, suggesting significant under prescribing of these important medications [11]. Similarly, Yong and colleagues studied 90,909 adults with persistent asthma and observed that only 47% were using more controller medications than quick-relief medications, and those who did were 27% less likely to have an asthma exacerbation [12]. This suggests that fostering increased patient-provider communication of asthma management has the potential to significantly improve asthma control.

One intervention strategy that has proven effective in improving the care that patients receive is reminding patients to ask their providers specific questions that lead to changes in their care [13–15]. This strategy is effective because patients who ask for tests and treatments tend to receive them [16, 17]. While a number of studies have observed that this method is effective in improving preventive services, such as immunizations, mammography, and colon cancer screenings [13], it has also been successful in improving a range of mental and chronic illnesses. For example, Kravitz and colleagues observed that standardized patients trained to portray a patient with major depression were more than twice as likely (76.0% v. 31.2%) to receive an antidepressant if they were coached to ask for one [16]. Additionally, previous studies by our research team have demonstrated that using web-based tailored messages can encourage patients to ask their provider specific questions to improve the care of chronic illnesses, such as migraine [18], osteoarthritis [19], hypertension [20] and asthma [21]. However, this approach has not been widely studied to understand its impact on asthma control. Given the power of disseminability through use of the Internet, further research of online tools to improve patient-provider communication and asthma self-management is warranted.

We sought to test the efficacy of an online tool designed to improve asthma control by reminding patients to ask their providers for changes to their treatment and to encourage specific self-care behaviors. The primary hypothesis was that participants randomized to the web-based intervention would have better asthma control, as measured by the Asthma Control Test (ACT), a 5-item validated self-report instrument [4,22,23]. The secondary hypothesis was that participants randomized to the intervention condition would receive a greater number of controller medications, thus demonstrating their improved asthma control.

## Methods

### Study design

The study design was a 12-month single-blind randomized controlled trial with 1:1 allocation ratio comparing the impact of an online tool designed to encourage patients to ask their provider questions about their asthma care versus a control intervention designed to encourage patients to ask their provider questions about preventive services unrelated to asthma control (e.g., cancer screening).

Randomization was performed after participants completed baseline self-report assessments online by linking an enrolled participant to the next available random allocation sequence; a computer-generated algorithm in the management module designed by study website programmers. Participants were blinded to their assignment.

All study participants were asked to use the online tool at least once each month, in which they would answer 10–15 series of questions about their health and health care. Based on their answers and pre-written rules, the online tool provided tailored feedback reminding patients to ask providers specific questions about their asthma medications and asthma self-care, to improve adherence to the 2007 NAEPP treatment guidelines [7]. The study was approved by the Penn State Hershey Institutional Review Board, IRB#28190EP (Study Protocol in S1 Protocol). As no face to face visits were performed during the study, informed consent was completed verbally by phone and participants electronically signed a data sharing agreement between Penn State Hershey Medical Center and their insurer, Highmark Blue Shield. The website was developed internally by the Department of Public Health Sciences at Penn State College of Medicine and all data was secured per security requirements of the Health Insurance Portability and Accountability Act (HIPAA).

### Participants and procedures

Participants were members of a large insurer, Highmark Blue Shield, with over 4 million members. Recruitment letters were sent to members who were: 1) enrolled in Highmark Blue Shield for at least one year, 2) between the ages of 21–60 years and 3) met Healthcare Effectiveness Data and Information Set (HEDIS) criteria for persistent asthma [24]. The HEDIS criteria identify individuals with persistent asthma based on their pattern of medication use specific to asthma (e.g., albuterol), emergency room visits or hospitalizations with a principal diagnosis of asthma, and outpatient visits coded by the provider with a diagnosis of asthma [24]. Potential participants were excluded if, during a phone screener, they reported never receiving a diagnosis of asthma from a health care provider, were not able to read and speak English fluently, did not have Internet access at home or work or were pregnant. In addition, those with a history of more than 20 pack-years of cigarette smoking were excluded, as many of these individuals have chronic obstructive pulmonary disease rather than asthma [25]. Enrolled participants were provided a secure login and password for using the online tool. They were compensated financially for completing data collection at baseline, 6 and 12 months. Recruitment began in 2009 and all follow-up measures were completed by 2012.

### Intervention condition (IC)

Participants in the IC were asked to use the online tool at least once every 30 days and within 14 days of their next scheduled health care provider visit, though they could use it any time. During each website use, IC participants answered a series of questions (11 total) about their asthma symptoms (e.g., rescue inhaler frequency, night symptom frequency), availability of oral corticosteroids at home for exacerbations, and asthma care received from providers, such as an asthma management plan (Table 1). Additionally, participants entered the names of all of their current asthma medications from a pick-list of medications from the National Drug Code (NDC) Directory [26], reported the number of days each week that each medication was used, and identified if any medicines bothered them. Finally, participants were asked to update their tobacco use and pregnancy status (if applicable) as well as record their next scheduled visit with their asthma care provider (e.g. allergist/pulmonologist).

**Table 1:** Monthly intervention asthma questions.

VARIABLE	QUESTIONS (NAME: question)	Error Checking	Who is asked	RESPONSES
HEADER1	Please answer the following questions. Your answers will help us to suggest questions to ask your doctor at your next visit. For each answer requiring a date, please select the month and year to the best of your knowledge.	N/A	N/A	none, just a header
DAYSX	In the past 7 days have you: Had daytime asthma symptoms more than 2 times?	Required	All	Yes (1) No (2)
LIMITSX	In the past 7 days have you: Had your activity or exercise limited by asthma?	Required	All	None of the time (1) Some of the time (2) All of the time (3)
NIGHTSX	In the past 7 days have you: Woken up at night because of asthma?	Required	All	Yes (1) No (2)
RESCUE	In the past 7 days have you: Used an inhaler for quick relief of asthma symptoms more than two times?	Required	All	Yes (1) No (2)
EXACERB	Over the past few days, how has your asthma been?	Required	All	Much better than usual (1) About the same as usual (2) Much worse than usual (3)
ORALCS	Do you have a medication at home that you can take by mouth for a few days when you asthma is worse than usual? Typically these are steroid pills, such as prednisone.	Required	All	Yes (1) No (2)
ED	During the past 12 months, have you had to go to the emergency room or urgent care center because of your asthma?	Required	All	Yes (1) No (2)
TRIGGERM	When was the last time a doctor, nurse or other health professional evaluated you to determine whether your asthma symptoms could be improved by reducing your exposure to an allergen or irritant at home or at work?	Fields be prepopulated at future visits	All	m: 1–12, missing y: 1955 –present, missing
TRIGGERY				
TECHM	When was the last time a doctor, nurse or other health professional watched you using your inhaler to make sure that you were using it correctly?	Fields be prepopulated at future visits	All	m: 1–12, missing y: 1955 –present, missing
TECHY				
SPECM	When was the last time you saw an asthma specialist? These doctors are often called "allergists" or "pulmonologists.”	Fields be prepopulated at future visits	All	m: 1–12, missing y: 1955 –present, missing
SPECY				
AAP_EXAC	Do you know what you are supposed to do when your asthma is getting worse?	Required	All	Yes (1) No (2) Not Sure(8)

The online tool then provided tailored feedback to remind patients to: 1) ask health care providers specific questions that may improve asthma control (e.g., additional controller medications) and 2) consistently perform specific self-care behaviors (e.g., proper inhaler technique). To create the intervention, a general internist (CS) worked with a specialist (TC) to review the 2007 NAEPP Guidelines and identify recommendations that were based on the strongest evidence and could be influenced by patient reminders [7]. Once the actionable recommendations were identified, questions were identified to measure adherence to the recommendation and then feedback was written to remind patients what questions they may want to ask their provider at an upcoming visit as well as what self-care they should be more adherent to, based on the guidelines. The feedback also included a layperson-oriented explanation of why the questions were important to ask of their asthma care provider, as well as a link to an external website that supported the recommendation (i.e., National Heart, Lung and Blood Institute, www.nhlbi.nih.gov). See Table 2 for examples.

**Table 2:** Sample guideline recommendations, rules and feedback.

NAEPP Recommendation	Rule for including question	Feedback for those not adherent to recommendation
Daily long-term control medication is recommended for patients who have persistent asthma (page 334)	Asthma is not controlled (e.g., rescue inhaler used > 2 times weekly or night symptoms) and medication list does not include inhaled corticosteroid (ICS).	WOULD I BENEFIT FROM USING AN INHALED STEROID TO CONTROL MY ASTHMA? You are not using an inhaled steroid medicine for your asthma. These medicines are the best for controlling asthma symptoms. Two common names are Aerobid or Flovent. Click here for more information.
Recognition of early signs of worsening asthma and taking prompt action (page 373)	Asthma is not controlled (e.g., rescue inhaler used > 2 times weekly or night symptoms) AND no provider visit scheduled in next 6 weeks.	YOUR ASTHMA IS NOT CONTROLLED AND YOUR VISIT IS NOT SOON ENOUGH. From what you told us, your asthma is not controlled, but you’re not seeing your doctor for more than 6 weeks. You may want to call schedule a visit to see your doctor sooner or call your doctor. Click here for more information.
Add a long-acting beta-agonist (LABA) to a low-dose of inhaled corticosteroid ICS (page 343).	Asthma is not controlled (e.g., rescue inhaler used > 2 times weekly or having night-time symptoms) AND medication list includes an inhaled corticosteroid but no LABA.	WOULD I BENEFIT FROM USING A LONG-ACTING INHALER TO CONTROL MY ASTHMA? Since you are using an inhaled corticosteroid, your doctor may decide to start a second medication. The best choice for a second medicine would probably be a long-acting inhaler like Serevent, or your doctor may change you to a single medicine that has a corticosteroid and a long-acting inhaler, such as Advair or Symbicort. Click here for more information.
Monitor the following factors at each visit: patient’s adherence to the regimen, inhaler technique, and side effects of medications (page 63)	Asthma is not controlled (e.g., rescue inhaler used > 2 times weekly or having night-time symptoms) AND as not been observed for inhaler technique in past 12 months.	AM I USING MY INHALER CORRECTLY? Using an inhaler correctly is not simple. Most people make mistakes with how they use it. For example, many people don’t fully exhale before triggering the inhaler. And many people don’t hold their breath for 5–10 seconds after breathing in the medicine. Ask your doctor or someone else in the office to watch you use your inhaler. Click here for more information.

When using the website routinely each month, IC participants clarified the date of their next asthma care provider visit. In keeping with the 2007 NAEPP Guidelines [7], a reminder email was sent to participants to use the website before doctor visits. In addition, the reminder email suggested that participants make an earlier visit if their asthma was not controlled and their next scheduled visit was greater than two weeks in the future. As the intervention was mainly designed to be used before a visit with the participant’s asthma care provider, we tracked the dates of upcoming asthma care provider appointments and actively reminded participants to use the website before these visits. We assumed that the intervention would be less effective if used long before or following an office visit, as the intervention was designed to “activate” patients to ask specific questions during visits with their asthma care provider to improve clinical inertia. For that reason, participants in both conditions received automated email reminders at the beginning of each new 30 day cycle to log in to the interactive website each month. If the participant did not use the intervention by day 20 of each 30 day cycle, they received an automated reminder email to use the intervention. Additionally, to ensure completion of study measures, a reminder email and a reminder phone call from study staff took place if baselines measures were not completed within 14 days of the 30 day cycle in which the measures were due. For the 6 and 12 month measures, a reminder email and reminder phone call from study staff took place if measures were not completed within 25 days of the 30 day cycle in which they were due.

### Control condition (CC)

The control condition was designed as an active treatment control that asked questions and gave feedback about preventive services (e.g., colon cancer screening) that would be unlikely to change asthma care. This control condition design was chosen to limit attrition and control for contact time. As patients with chronic medical conditions, including asthma, are less likely to receive preventive services such as pap testing and mammography [27–29], we chose an active treatment control condition that met a need of patients with asthma, but would not likely modify asthma control. We used the United States Preventive Services Task Force (USPSTF) guidelines to determine expected screenings and determine questions patients could ask providers about tests they may be due to receive [30].

### Measures

Asthma control was assessed using standard instruments at baseline, 6 months, and 12 months. The primary outcome measure of asthma control was the 5-question Asthma Control Test (ACT), a validated and commonly used self-report survey of asthma control [22,31]. Asthma control was also assessed using six items from the CDC’s National Asthma Survey (NAS) [32,33]. Five items from the NAS were also used to assess asthma-related care that may have been influenced by the intervention, such as “*Has a doctor or other health professional ever taught you how to use a peak flow meter*?” [32,33].

At the end of the study, Highmark Blue Shield provided Penn State researchers with insurance claims data for all enrolled participants for two years prior to randomization and 18 months post-randomization. Asthma-related claims for hospitalization, outpatient visits, emergency department visits and medications were examined. Data sharing agreements to allow this process were signed by each participant during the informed consent process and approved by the Penn State College of Medicine Institutional Review Board. The Medication Possession Ratio (MPR), a commonly used measure of adherence that is predictive of asthma exacerbations, was calculated based on the percentage of days that each medication was available to participants [34–36].

Age, gender, race, ethnicity, educational attainment, marital status and insurance status were measured, using standardized instruments such as those from the Behavioral Risk Factor Surveillance System (BRFSS) [37,38]. These variables have been shown to be related to asthma prevalence, asthma quality of care and asthma-related quality of life [39–41].

### Statistical analysis

All analyses were carried out using SAS Software 9.3 (SAS Institute, Cary, NC). Sample size calculations found that we would need to recruit 408 patients to have an 80% power and alpha of 5% (0.05) and a two-tailed test to detect a difference of 70% vs. 55% between intervention and control group. Categorical variables were summarized with frequencies and percentages, while continuous variables were summarized with means, standard deviations, medians, and quartiles. The distribution of continuous variables was evaluated using box plots, histograms, and normal probability plots. Cut-points were chosen for reporting results based on recommendations from the NAEPP Guidelines. For example, scores for the question “*In the past 30 days*, *on how many days have you had symptoms of asthma*?” were divided into ≤ 8 v. >8 as the NAEPP guidelines include a goal that asthma should cause symptoms on no more than 2 days each week [7].

For demographic variables and other characteristics measured at baseline, comparisons were made between treatment groups using a two-sample t-test with means for continuous variables and a Chi-square test with percentages for categorical variables. An exact test was used as needed for cell counts too small for the Chi-square test to be valid. The same methods were used to test for differences in the baseline characteristics between participants who completed the study and those lost to follow-up.

In making comparisons within and between treatment groups at baseline and 12 months, we followed intention-to-treat (ITT) principles by using all of the available data in the models [42]. The only variable that differed significantly between conditions at baseline was the percentage of individuals in each group who were denied insurance in the past 12 months, so this variable was added as a covariate to all models. For continuous outcome variables, a linear mixed effects model was employed, and differences were quantified with means. For binary outcome variables, a Generalized Estimating Equations (GEE) model was utilized, and differences were quantified with percentages and odds ratios. To determine the impact of losses to follow-up on the results, we conducted a sensitivity analysis using a more conservative approach that replaced missing data with data from the last visit carried forward (LOCF) [42]. These sensitivity analyses revealed similar results to the analyses without replacement, so the results are presented in their original, non-replaced, form.

## Results

### Recruitment

**Fig 1:**
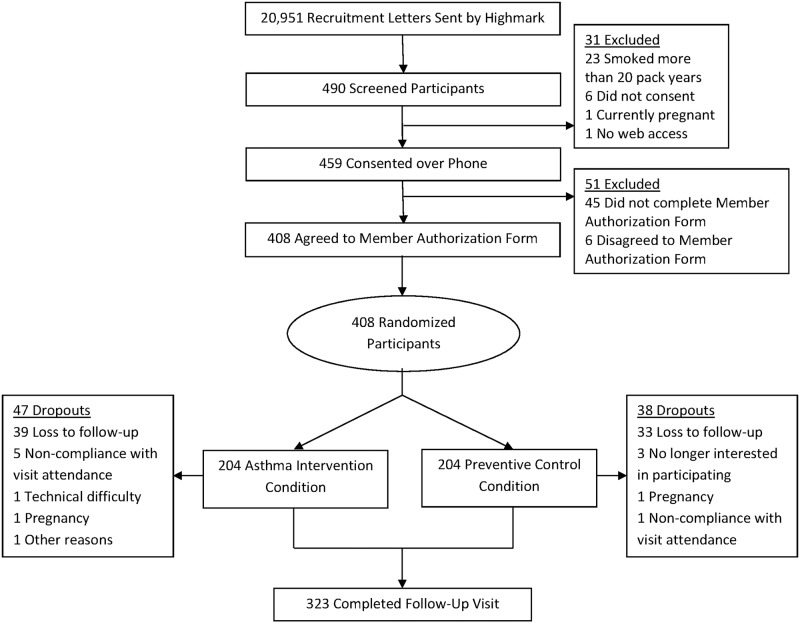
CONSORT.

Highmark sent recruitment letters to 20,951 individuals who met criteria for age and persistent asthma. Of the individuals who responded to the letter, 408 met inclusion criteria and were randomized. After 12 months, 323 (79.2%) participants completed the 12 month follow-up measures (Fig 1).

### Patient characteristics

Table 3 lists demographic variables and other characteristics measured at baseline. The only significant difference between groups at baseline was the percentage of individuals who reported “*a time in the past 12 months when you needed medical care but could not get it*” (2.0% vs 6.9%; *p* = .016). The average age of participants was 47.4 years and the average body mass index (BMI) was 30.8. Most participants were female (61.9%) and white (84.2%). A small proportion of participants smoked at baseline (3.2%), but a larger proportion reported smoking more than 100 cigarettes in their lifetime (27.1%).

**Table 3:** Baseline characteristics.

	Total (N = 407)	Intervention (N = 203) †	Control (N = 204)	P-value*
Demographics				
Age, Mean ± SD	47.4 ± 9.3	47.6 ± 9.1	47.2 ± 9.6	0.65
BMI, Mean ± SD	30.8 ± 7.6	31.0 ± 7.6	30.7 ± 7.7	0.70
Gender, Female, %	61.9	63.6	60.3	0.50
Race: White, %	84.2	81.7	86.8	0.16
Ethnicity: Hispanic, %	3.4	3.9	2.9	0.58
Married, %	73.7	75.4	72.1	0.45
Education: College 4+ years, %	58.5	58.1	58.8	0.89
Employed for wages, %	76.9	74.4	79.4	0.23
Income <$50,000, %	41.2	41.8	40.5	0.79
Health: Very good/Excellent, %	46.4	44.3	48.5	0.40
Denied Insurance In Past 12 Months, %	4.4	2.0	6.9	0.02
Have One Person Thought of as Personal Doctor, %	93.8	92.5	95.1	0.29
Current Smoker, %	3.2	3.5	2.9	0.77
Smoked More Than 100 Cigarettes Lifetime, %	27.1	25.1	29.1	0.37

^†^ One randomized subject dropped out prior to the baseline visit and is not included in the Intervention group.

* P-values from Two-sample T-test (Mean ± SD) or from Chi-square test (%), exact test used when needed.

### Fidelity

**Fig 2:**
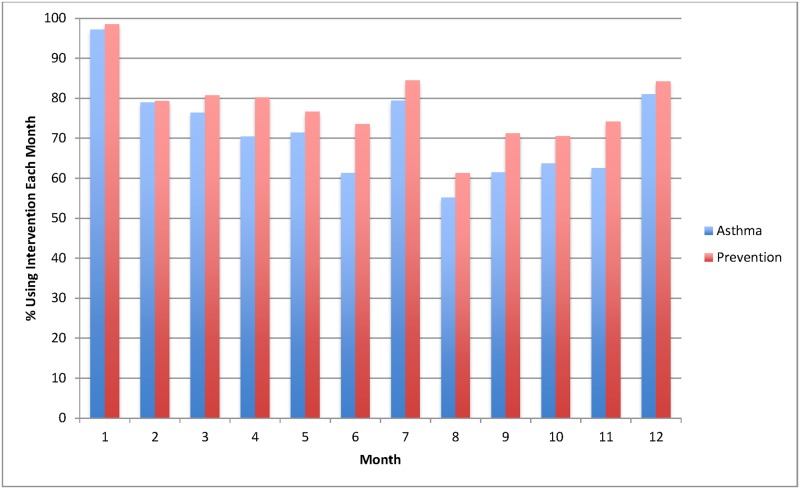
Fidelity: Percentage of participants using intervention at least once in each month.

The percentage of participants in each condition who used the website each month can be seen in Fig 2 below. While fidelity varied month to month, over the course of the 12 month study, IC participants used the intervention in 76.7% of months v. 83.8% for CC participants, a non-significant difference (Fig 2).

### Outcome measures

As seen in Table 4, the main outcome measure, the Asthma Control Test (ACT), improved more in the IC than CC (+2.3 v. +1.2, p = 0.02). Each individual item of the ACT showed improvement favoring the IC, but none of these differences was significant. In addition, 4 of 6 asthma control questions from the National Asthma Survey (NAS) showed greater improvement in the IC, but none of these were significant.

**Table 4:** Asthma control and quality of life outcomes based on the Asthma Control Test (ACT), National Asthma Survey (NAS) and National Asthma Education and Prevention Program (NAEPP).

Outcome †	Intervention (N = 157)*			Control (N = 168)*			P-value
	Baseline	12 Months	Change from Baseline	Baseline	12 Months	Change from Baseline	
**Asthma Control Test (ACT)**							
Asthma Control Test (ACT) Score	17.7 (16.7, 18.6)	19.9 (18.9, 20.9)	2.3 (1.6, 2.9)	17.9 (17.0, 18.8)	19.1 (18.2, 20.0)	1.2 (0.6, 1.8)	0.02
ACT Score ≥ 20 (%)	49.8	72.6	22.8	52.9	66.7	13.8	0.13
In the past 4 weeks, how much of the time did your asthma keep you from getting as much done at work or at home? (“a little of the time”+ “none of the time”, %)	78.8	89.8	11.0	86.8	91.1	4.3	0.30
How would you rate your asthma control during the past 4 weeks? (“well controlled” + “completely controlled”, %)	58.1	73.9	15.8	63.2	71.4	8.2	0.17
During the past 4 weeks, how often have you used your rescue inhaler or nebulizer medication)? (“once a week” + “once or twice”+ “not at all”, %)	49.8	73.3	23.5	46.1	60.1	14.0	0.12
In the past 4 weeks, how often have you had shortness of breath? (“once or twice a week” + “not at all”, %)	62.1	83.4	21.3	63.2	78.0	14.8	0.13
During the past 4 weeks, how often did your asthma symptoms wake you up at night or earlier than usual in the morning? (“once or twice a week” + “not at all”, %)	70.0	89.2	19.2	76.0	82.7	6.7	0.01
**Asthma Control, National Asthma Survey (NAS)**							
In the past 30 days, on how many days have you had symptoms of asthma? (≤8, %)	56.7	77.1	20.4	58.8	73.8	15.0	0.32
During the past 30 days, on how many days did symptoms of asthma make you limit your activity? (0, %)	48.8	66.2	17.4	40.7	60.7	20.0	0.72
During the past 30 days, on how many days did symptoms of asthma make it difficult for you to stay asleep? (≤2, %)	69.2	82.2	13.0	75.0	82.7	7.7	0.40
In past 3 months, how many asthma attacks have you had? (0, %)	37.3	56.7	19.4	36.8	53.6	16.8	0.67
During the past 12 months, have you had to go to the emergency room or urgent care center because of your asthma? (No, %)	88.4	90.5	2.1	87.2	94.6	7.4	0.12
In the past 7 days have you had daytime symptoms more than 2 times? (No, %)	53.8	73.9	20.1	53.2	64.7	11.5	0.12
**Asthma Care, National Asthma Survey (NAS)**							
Has a doctor or other health professional ever taught you how to recognize early signs or symptoms of an asthma episode? (yes, %)	62.0	76.4	14.4	64.9	74.9	10.0	0.50
Has a doctor or other health professional ever taught you what to do during an asthma episode or attack? (yes, %)	75.5	85.4	9.9	71.3	80.8	9.5	0.81
Has a doctor or other health professional ever taught you how to use a peak flow meter? (yes, %)	67.0	75.2	8.2	67.8	73.1	5.3	0.33
Has a doctor or other health professional ever given you an asthma management plan? (yes, %)	16.0	29.3	13.3	20.3	31.7	11.4	0.53
Have you ever taken a course or class on how to manage your asthma? (yes, %)	3.5	9.6	6.1	5.9	12.6	6.7	0.74

^†^ %, P-value testing for difference in change from baseline between Intervention and Control groups from Binary Generalized Estimating Equations (GEE) model adjusted for denial of insurance.

* Mean (95% CI), P-value testing for difference in change from baseline between Intervention and Control groups from Linear Mixed Effects Model adjusted for denial of insurance

Note: Numbers differ for survey completion: 326 participants provided data on medications, 325 participants completed ACT and NAS, and 324 participants provided data for the NAS.

No differences were noted in the percentage of individuals receiving care consistent with the NAEPP guidelines, using items from the NAS (Table 4). For example, there was a 14.4% increase in the percentage of participants in the IC who were taught by a health professional to “*recognize early signs or symptoms of an asthma episode*” versus a 10.0% increase in the CC (p = .50). Also, no significant differences were observed in asthma-related healthcare utilization or medication adherence, measured by the Medication Possession Ratio (MPR), from claims data obtained by Highmark (Table 5). Participants in both conditions reported increases in usage of all asthma medications, including controller medications, but these differences were not significant.

**Table 5:** Secondary outcomes: Asthma-related healthcare utilization or medication adherence, based on insurance claims.

Outcome	Intervention (N = 158) *			Control (N = 168) *			P-value^†^
	Baseline	12 Months	Change from Baseline	Baseline	12 Months	Change from Baseline	
Number of Asthma Medications, Medications*	1.72 (1.40, 2.04)	2.14 (1.82, 2.46)	0.42 (0.26, 0.58)	1.69 (1.39, 1.99)	1.93 (1.64, 2.23)	0.25 (0.09, 0.40)	0.12
Number of Asthma Controller Medications	1.16 (0.91, 1.41)	1.34 (1.09, 1.60)	0.18 (0.07, 0.30)	1.05 (0.82, 1.28)	1.17 (0.94, 1.41)	0.12 (0.01, 0.24)	0.46
Percentage using at least one Asthma Controller Medication	70.9	81.0	10.9	71.6	81.6	10.0	0.95
Number of Emergency Room Visits	0.23 (-0.08, 0.54)	0.03 (-0.28, 0.23)	-0.26 (-0.44, -0.08)	0.12 (-0.17, 0.41)	0.04 (-0.20, 0.28)	-0.08 (-0.26, 0.10)	0.17
Number of Outpatient Visits	3.09 (2.45, 3.72)	2.96 (2.31, 3.61)	-0.13 (-0.54, 0.28)	2.80 (2.21, 3.38)	2.80 (2.19, 3.40)	0.0 (-0.41, 0.40)	0.67
Medication Possession Ratio (MPR) for all asthma medications	75.6 (70.5, 80.6)	77.8 (72.6, 83.0)	2.2 (-1.3, 5.7)	76.8 (72.1, 81.5)	80.1 (75.2, 84.9)	3.3 (-0.2, 6.8)	0.68
Medication Possession Ratio (MPR) Asthma Controller Medications	77.5 (72.1, 82.8)	79.4 (73.9, 84.9)	1.9 (-2.0, 5.8)	79.2 (74.2, 84.3)	82.7 (77.5, 87.9)	3.5 (-0.4, 7.4)	0.57
Medication Possession Ratio (MPR) Reliever Medications Only	69.4 (60.4, 78.5)	69.9 (60.2, 79.7)	0.50 (-7.2, 8.2)	69.2 (60.6, 77.8)	69.2 (59.9, 78.5)	0.0 (-7.5, 7.6)	0.93

^†^ %, P-value testing for difference in change from baseline between Intervention and Control groups from Binary Generalized Estimating Equations (GEE) model adjusted for denial of insurance.

* Mean (95% CI), P-value testing for difference in change from baseline between Intervention and Control groups from Linear Mixed Effects Model adjusted for denial of insurance.

## Discussion

In this randomized controlled trial, we tested the impact of using online patient reminders designed to help patients with persistent asthma ask specific questions during office visits and modify their self-care, in order to improve their asthma control. Each reminder was based on a specific recommendation in the 2007 NAEPP Guidelines [7]. Despite the brevity and simplicity of the intervention (~10 minutes monthly + minimal interactivity), participants randomized to the IC significantly improved their asthma control versus participants in the CC. Intervention participants increased their asthma control, as measured by the Asthma Control Test (ACT) by 1.1 [+2.3, 95% CI (1.6, 2.9) v. +1.2 (0.6, 1.8)] versus controls and had non-significant improvements in 9 of 11 individual asthma control survey items. The magnitude of the effect of using the online tool is similar to that of adding an additional controller medication [43]. We did not, however, observe differences in asthma utilization, such as emergency department (ED) visits or outpatient visits, though the rates of ED utilization (2.3% in the IC and 1.2% in the CC) were low at baseline.

Although the finding of improved asthma control was both statistically and clinically significant in the IC, the mechanism for the difference is not clear. We hypothesized that the difference would be due to an increase in the number of, or adherence to, asthma controller medications, though we observed no such differences due to the treatment. We speculate that the differences may have been due to other reminders included in the feedback, such as reminders about inhaler technique (See Table 2). Participants in the IC who had not been counseled about proper inhaler technique were encouraged to ask “*Am I using my inhaler correctly*?” and the online reminder suggested specific changes to technique: “*For example*, *many people don’t fully exhale before triggering the inhaler*. *And many people don’t hold their breath for 5–10 seconds after breathing in the medicine*” and included a link to another source of information about proper technique. Other reminders, based on recommendations from the NAEPP guidelines [7], focused on quitting smoking, controlling allergies and having an asthma management plan, also known as an Asthma Action Plan. The intervention, therefore, may have functioned primarily by improving asthma self-management behaviors, rather than by intensifying asthma medications. A future study should include a measure of inhaler technique to understand if this change mediated the improvement in asthma control.

This study supports the results of previous studies that have found that web-based asthma modules may improve asthma control and quality of life [44, 45]. However, there are important differences between our study and previous studies which highlight the strength of our intervention. For example, van der Meer, et al. also provided group education to their web-based self-management program [44]. Our intervention, which was strictly web-based, allows for increased dissemination to patients with asthma or other chronic conditions, given that 85% of adults use the Internet and 72% of Internet users look online for information about a specific disease or medical problem [46]. Additionally, Rasmussen and colleagues’ study focused more on web-based monitoring of asthma symptoms compared with specialist- or physician-monitored [45], whereas we provided tailored feedback that was meant to improve patient activation when meeting with a provider. Given that 74.5% of participants were satisfied or very satisfied with the feedback they received, it is reasonable to assume that patients may benefit from using this simple, online tool to improve asthma control. An additional strength of this study was the use of an active treatment control condition, where participants were given feedback on preventive services rather than asthma treatment. This was implemented to improve participant adherence and control for contact time while still providing participants with useful medical information and encouraging them to consider scheduling certain medical tests. We believe that a 12-month follow up completion rate of 79.7% indicated that we achieved success with this study design, as shown in prior studies [20,47,48]. Further, our study had a larger study sample than these two studies. Similarly, many studies have focused their web-based interventions on children [49, 50], whereas we chose to focus on adults.

Despite a single-blinded, experimental study design, high rates of fidelity and an active treatment control condition, the study has a number of limitations. First, not all clinically relevant outcome measures, such as pulmonary function, were measured. Second, measures of inhaler technique and audio recordings of provider interactions were not included and may have better enabled us to understand the reasons for the positive findings. Finally, the study enrolled patients of relatively high socioeconomic status (SES), who had both Internet access and health insurance, though asthma prevalence and severity are highest among individuals of lower SES [1]. While nearly 90% of US adults have Internet access via multiple modalities (e.g., computer, smartphone), access is still more limited among the elderly, poor, and those with a lower educational level [51]. Future research is needed to determine the best approach to utilize to reach these groups so they may receive feedback on their chronic medical conditions.

## Conclusions

A simple and brief online intervention improved asthma control, as measured by the Asthma Control Test, among insured patients in Pennsylvania. Although future studies are needed to understand the mechanism of the improvement, the magnitude of the effect was similar to the addition of an additional controller medication [43]. Given that half of US adults have a chronic illness [52] and that chronic illnesses account for 75% of health care costs [2], it is critical to identify simple tools to help patients in their care and improve outcomes. Online tools have the potential to be used in other conditions, are relatively cost-effective to disseminate and, as observed in this study, may have a significant impact on improving chronic disease management and the public’s health.

## Supporting information

S1 Checklist Consort checklist. (DOC)

S1 Protocol Study protocol. (DOC)

S1 Table Secondary outcomes by asthma severity. (DOCX)

S1 Appendix Histograms and box plots for each continuous outcome. (DOC)
