# Supplementary material for: Impact of online patient reminders to improve asthma care: A randomized controlled trial
Source: PLoS One. 2017 Feb 3;12(2):e0170447. doi: 10.1371/journal.pone.0170447 (PMC5291361; doi:10.1371/journal.pone.0170447)
Supplement: S1 Protocol — (DOC) [file pone.0170447.s002.doc]

**S1 Protocol. Study Protocol.**

**Improving the Quality of Asthma Control Using the Internet**

**Research Proposal**

## Specific aims or objectives

In the US, asthma affects approximately 15 million people, including 5 million children ^1^. The direct plus indirect costs of asthma, including lost productive time in the US are estimated to be greater than 12 billion dollars annually ^2,3^. Of this, at least 2.5 billion dollars of lost productivity is estimated to be due to the suboptimal care that patients with asthma receive ^4^. Despite widespread dissemination of evidence-based guidelines, more than half of adults with asthma have uncontrolled asthma ^5,6^. For example, Schatz and colleagues noted, among a random sample of adult patients with asthma who had health insurance, 51.7% had uncontrolled asthma ^5^. This lack of control is seen despite a large increase in the percentage of adults with asthma who use a controller medication ^7,8^. In addition to poor asthma control, several recommendations from the National Asthma Education and Prevention Program (NAEPP) guidelines, published since 1991, have not been consistently followed. The 2003 National Asthma Survey, for example, observed that only 55% of adults with asthma were taught to recognize early symptoms of asthma, only 47% were instructed to change their environment to improve asthma control and only 33% had ever received an asthma action plan ^9,10^. Though patient non-adherence to controller medicines likely contributes to poor asthma control, lack of asthma control likely reflects a widely observed tendency of physicians to make no changes in the face of patients who have not met a treatment goal for a chronic illness, known as “clinical inertia” ^11-15^. One intervention strategy that has proven effective in improving care is prompting patients to ask their providers specific questions that lead to changes in their care ^16-20^. This strategy is effective because patients who ask for tests and treatments tend to get them ^21,22^. This strategy has been widely successful in studies aimed at improving preventive care (e.g., pap testing), though its effects on improving chronic illness care, including asthma, have not been studied ^16,23^. Without an intervention, however, patients do not know the questions to ask that will lead to improved chronic illness care ^24^. To test the efficacy of this approach for improving asthma care, we have developed an interactive website to be used by patients with asthma at least once each month, during exacerbations and before asthma care provider visits ^25^. The program asks patients questions about their asthma symptoms, disability from asthma, the names and doses of asthma medicines and about the care they’ve received for asthma. Based on responses to these questions and decision rules from the 2007 NAEPP Guidelines ^26^, patients are given personalized feedback. The feedback includes questions that the patient should consider asking during their next doctor visit, along with a lay explanation of why they should ask each question and links to reputable websites (e.g., www.nhlbi.nih.gov) to learn more about each suggested question. Sample questions that patients are encouraged to ask include: “Would I benefit from using an inhaled corticosteroid to control my asthma?”, "What medicine change would help to control my asthma?” and “Should I have a prescription for steroid pills to have at home if my asthma worsens?”. The interactive website is designed to activate patients to ask questions that may improve the quality of care they receive for asthma. Previous studies have shown that the interactive website leads to important changes in what happens during doctor visits, that both physicians and patients find the interactive website useful and empowering to patients and that the feedback does not harm the doctor-patient relationship ^25,27,28^. In order to build upon those positive findings, we propose an efficacy study to test the effect of this approach for improving asthma care and control. We will enroll 408 adult patients from a large health plan in Pennsylvania and randomize them to use the asthma module (IC) or the adult preventive services module (CC) of the interactive website for a period of 12 months. Primary Hypothesis: is to determine the efficacy of having patients use the asthma module of the interactive website to improve asthma control, based on the Asthma Control Test (ACT). After 12 months, we hypothesize that 70% of patients in the IC condition will have their asthma controlled (ACT ≥ 20) versus only 55% of patients in the CC condition. Secondary Hypothesis: is that at the end of 12 months, we hypothesize that IC subjects, compared to CC subjects, will have greater improvements in: a) asthma-related emergency department visits, b) asthma related quality of life, c) pulmonary function, d) controller/reliever medication ratio, e) satisfaction with asthma treatment, f) adherence to their asthma controller medications and f) self-management support counseling. We will also calculate the relative cost-effectiveness, from the perspective of society and health plans, of providing patients with access to the interactive website.

## II. Background and Significance

Asthma morbidity and mortality. Asthma is a chronic inflammatory disease of the lungs. In the United States, asthma affects approximately 15 million persons, including 5 million children ^1^. It is estimated that people in the United States with asthma collectively experience 100 million days of restricted activity, nearly 500,000 hospitalizations and approximately 5,000 deaths annually ^29^. In addition, patients with asthma report significantly more physically unhealthy days, more mentally unhealthy days and more days with activity limitation than those without asthma ^30^. Despite widespread availability of consensus guidelines from the National Asthma Education and Prevention Program, many patients with asthma have poor asthma control and do not get care consistent with the guidelines ^26^. For example, Mularski and colleagues recently published a study showing that adult asthma patients received 53.5% of recommended care processes ^31^. Progress has been made with improving the use of controller medications in the United States, however. In a study of the National Ambulatory Medical Care Survey, Ma and colleagues observed that inhaled corticosteroid use in asthma increased from 25% in 1992 to 42% in 2002 ^8^. Similarly, among a national group of health plans, controller medication use was up to over 80% in 2005, versus only 58% in 1997 ^7,8^. Despite this increase in availability of controller medicines, such as inhaled corticosteroids (e.g., beclomethasone) and long-acting beta-2 agonists (e.g., salmeterol) more than half of adults with asthma do not have their symptoms controlled ^5,6^. For example, Schatz and colleagues noted, among a random sample of asthmatic patients with health insurance, 51.7% had an Asthma Control Test (ACT) score < 20, a level at which the 2007 NAEPP Guidelines suggest is consistent with uncontrolled asthma ^5,26^. Similarly, Vollmer and colleagues reported that 52% of 5181 adults in a large managed care organization had at least one asthma control problem, according to the Asthma Quality of Life Questionnaire (AQLQ) ^6^. Carlton and colleagues reported that nearly 75% of 25,163 adults with asthma had at least one symptom consistent with poor control ^32^. A major reason for this lack of asthma control is that many patients whose asthma is not controlled are taking one, rather than multiple, controller medications, which are suggested in the 2007 NAEPP Guidelines ^26,32^. This suggests that focusing solely on the availability of controller medication use may not be enough to improve asthma control in the United States. Many other recommendations, however, are not being followed. The 2003 National Asthma Study, however, observed that fewer than 70% of adults with asthma were taught to recognize early symptoms of asthma, fewer than 50% were instructed to change their environment and fewer than 30% received an asthma action plan ^9^. Other investigators have observed similar patterns ^10,31,33^. In summary, this suggests that there are many opportunities to improve the care and control of asthma, by more rigorously applying clinical practice guidelines. This also suggests the need to modify the main outcome measure in the study, from controller medication availability, to asthma control. Many patients have at least one controller medication and still have poor asthma control.

Theoretical Model of Chronic Illness Care Interventions: The Chronic Care Model (CCM) summarizes the basic elements for improving chronic illness care and will, therefore, serve as the model for the planned intervention. The intervention will address several of the critical elements of the chronic care model, including 1) self-management support, 2) decision-support and 3) clinical information systems. The overall goal of the intervention, in keeping with the CCM, is to foster productive interactions between an informed, activated patient and physician ^34,35^. Preliminary results suggest that organizational interventions based on this model lead to improvements in the management of chronic diseases ^36,37^. Though predating the CCM, Greenfield and colleagues tested an intervention in which patients with diabetes met with a research assistant who reviewed their medical record and, using a diabetes care algorithm, encouraged the patient to use the information reviewed to negotiate medical decisions with the doctor ^38^. The intervention had a significant positive effect on glucose control and serves, along with the CCM, as a model for the design of the intervention to be tested. In 2005, a randomized controlled trial by Kravitz and colleagues, published in the Journal of the American Medical Association, showed that prompting patients to ask specific questions, thereby becoming “informed, activated patients” in keeping with the CCM, can impact the care that is delivered ^22^. The planned study is the first to our knowledge to examine the ability of patients to impact the quality of asthma care they receive by asking specific questions to their provider at the point of care.

Barriers to following clinical practice guidelines and changing physician behavior: There are many barriers to following clinical practice guidelines ^39-43^. For example, Cabana and colleagues noted that a lack of familiarity with the guidelines, a lack of awareness of the guidelines and inertia of previous practice were significant barriers ^11,44^. This inertia, known as “clinical inertia” has now been well described as a physician trait, leading to problems with the control of a range of chronic medical problems ^13,45-48^. Of the methods for modifying physician behavior, including peer review, opinion leaders, auditing, academic detailing and reminders, Smith concluded that "of all interventions, reminders show the best evidence to date of consistent effectiveness" ^49^. Such reminders, especially those delivered via computer at the point of care, have been successful for changing preventive care (e.g., pap screening) but, to date, less successful for changing chronic illness care ^19,50-55^.

Asthma quality of care improvement interventions: Asthma interventions typically are of three types. First, interventions designed to improve clinically-oriented patient outcome measures, such as asthma control, quality of life or hospital admission rates. Examples of such interventions include medications, patient education and self-management ^56-64^. Second, interventions designed to improve processes of care, such as the receipt of controller medications or asthma action plans. Examples of such interventions include case management, physician education or feedback and point of care reminders that are computerized or placed on flow sheets ^65-70^. Third, interventions that provide physicians with point of care reminders that suggest changes in care ^32,54,71-76^. Though point of care reminders (to both patients and providers) are consistently effective at improving preventive services (e.g., pap testing) ^16^, only four published studies to our knowledge have examined the effect of point of care reminders in asthma and none of the studies examined point of care reminders to patients. McCowan and colleagues observed that computerized reminders, directed to physicians, decreased the frequency of asthma exacerbations ^77^. Eccles and colleagues, however, found no effect of reminders to physicians on the quality of asthma care, such as prescribing inhaled corticosteroids or completing an asthma action plan with patients ^70^. The intervention studied by Eccles and colleagues, however, was felt to be too cumbersome to use in routine care by physicians, leading physicians to rarely use the software ^70^. Similarly, Tierney and colleagues observed no effect in the quality of care by using point of care computerized reminders to physicians and pharmacists, similar to the negative findings they observed in using physician-directed point of care computerized reminders to improve hypertension care ^72,78^. Porter and colleagues observed only a very small effect on controller medication prescription in asthma from an intervention that created reminders for physicians seeing asthma patients in the emergency department ^79^.

Limitations of Electronic Medical Record (EMR)-Based Reminder Systems: We chose not to base our intervention within an Electronic Medical Record system (EMR) for three reasons. First, though EMR use is growing, as of 2005 only 25% of outpatient visits were in offices using EMRs ^80^ and only a small percentage of these visits are with practices that have PHRs that allow patients to enter data. Patient data entry is necessary in this study because asthma care decisions are mostly made on the basis of patient symptoms ^26^. Second, EMR-based reminders to physicians are effective for improving preventive services, but are generally not effective for improving chronic disease management ^70,72,78,79,81^. Third, Internet access has grown to the point that at least 75% of Americans have internet access at home or work ^82,83^ . In summary, as our main goal in this study is to test the effect of giving personalized reminders to patients to improve chronic disease management, and few EMR systems allow patients to enter their symptom data, we chose to test the hypothesis using a web-based tool. If the study proves to have a positive effect, future PHRs can incorporate such tools for a wide range of conditions.

Consumer Demand for Health Information on the Internet and the Decreasing “digital divide.” A May 2005 report from the Pew Internet and American Life Project noted that 79% of adult Internet users in the US have searched for health and medical information and 66% have searched for information about a specific disease or medical problem ^84^. A 2006 survey noted that nearly 60% of those who experienced a loved one's recent health crisis say the single most important source of information was something they found online ^85^. This behavior cuts across racial and ethnic boundaries. For example, 58% of African-Americans with Internet access sought health information on the Internet ^86^. In addition, Internet access has continued to grow. Approximately three in four Americans have access to the Internet at home or work ^82,83^ and these increases have also been quite rapid among lower income and minority Americans ^87^. Though web-based interventions to improve asthma self-management exist ^56^ we have identified no interactive websites that help patients negotiate care decisions with their provider, in order to improve asthma control. This is exactly the need that our intervention seeks to address. These trends suggest that the Internet is rapidly growing toward ubiquity and that Internet-based health applications will have a large, diverse and receptive audience ^84,88,89^.

SIGNIFICANCE: There is a great need for improving asthma control in the US. Our review of the literature suggests that asthma control can be improved by applying evidence-based asthma guidelines. In particular, improving several quality “gaps” should be expected to improve asthma control, including: 1) continue to intensify treatments, by adding additional medications, increasing the doses of controller medications, making sooner return visits and making visits to see specialists, until asthma control is reached, 2) assess adherence and discuss adherence-related issues with a provider at each visit and 3) create an asthma action plan and have it reviewed regularly with a provider. We propose to test the effect of personalized reminders, based on the 2007 NAEPP Guidelines, to help patients negotiate these care decisions with their provider, in order to improve asthma control. We see the current project as building on our positive pilot studies of web-based tools to improve the quality of chronic disease management by leveraging the power, ubiquity and cost effectiveness of the Internet. Though reminders given to patients improves the delivery of preventive services (e.g., pap testing), no study to our knowledge has examined the potential of such prompts to patients to improve the control of asthma. As treatment decisions for patients with asthma, as opposed to most other conditions (e.g., hypertension), are typically based on patient-reported symptoms, we see asthma as an ideal condition in which to study the effect of personalized reminders to patients, based on information patients enter into a web-based tool. As the use of the interactive website does not require time and effort of office staff or health plan personnel, it may overcome many of the limitations of other commonly used quality improvement interventions. Should the intervention improve the control of asthma, we expect at least four parties to have an interest in using it: 1) patients, to learn what to expect and what questions to ask during their doctor visits, 2) health care providers, to assure that the care they deliver is consistent with consensus guidelines and “pay for performance” initiatives, 3) employers, to decrease lost productive time due to asthma and 4) managed care organizations, to show to employer clients that they have interventions in place to improve the quality of care they provide. Given the steady increase in Internet access, we believe that if the intervention proves to be effective, it may have a significant impact on the control of asthma, as well as on other chronic medical conditions.

## III. Research Design, Methods, and Human Subjects

### 1. Population to be studied (including control subjects)

Approximately 408 patients with asthma between the ages of 21-60 years old, who are members of Highmark.

### Inclusion/exclusion criteria

In order to be eligible for the study, all patients must:

1) be between 21 and 60 years of age

2) have evidence of physician-diagnosed asthma, including at least one documented asthma-related medical encounter in the past year AND at least 3 months of asthma-related medications in the past year.

3) have self-reported asthma, based on questions from the National Asthma Survey from the Centers for Disease Control (CDC) ^9^.

4) speak and read English fluently

5) have access to the Internet at home or at work

6) not have a history of more than 20 pack-years of cigarette smoking.

7) be enrolled in a Highmark health plan .

8) not currently pregnant

No children will be included in the proposed. While this work is important to do, we believe that the costs of running two parallel studies (two entirely different websites and recruitment strategies) would not outweigh the additional knowledge learned, at least at this time. As the studies that informed the creation of the intervention only included adults ^24,27,28,90^, a significant amount of development work would need to be done that would likely result in very different interventions for adults and children. If this approach is successful, future studies could extend these findings by developing interactive websites for children, who, on average, have higher rates of Internet access ^83^, or for their parents to use.

### 3. Study design

The study is randomized control trials that will be used to determine the efficacy of having patients utilize the asthma module of the interactive website to improve asthma control. Subjects will be randomized to either the intervention (asthma module) or control (preventive module) at the baseline visit.

Overall, the intervention is an interactive website. The website is designed to encourage patients to ask specific questions during visits with their asthma care provider that will help to improve the quality of their care and asthma control. IC subjects will use the asthma module of the interactive website for 12 months, which includes web-based asthma feedback, based on patient self-report. The feedback suggests questions to ask during visits with asthma care providers, reasons for asking the questions and links to other trustworthy websites that support the message. The website will be used a) each month and b) before visits with asthma care providers. When using the website routinely each month, the subject will report on their asthma control as well as clarify the date of their next asthma care provider visit. This allows the website to remind patients to use the website before doctor visits as well as to suggest that patients make a sooner visit, in keeping with the 2007 NAEPP Guidelines (page 345), if their asthma is not controlled and their next scheduled visit is far in the future.

Overall, the control condition is an active treatment control condition, in which subjects will be treated identically, except that the content of the questions and feedback on the interactive website will focus on adult preventive services (e.g., mammography screening, tetanus immunizations) rather than asthma. The control condition is an active treatment control condition designed to improve the use of adult preventive services (e.g., pap testing), but is unlikely to change asthma care. This control condition design was chosen to limit attrition and control for contact time, which will best enable us to isolate the effects of the intervention. Many similar studies have used this control group design ^91-93^. We considered several different control conditions, including a no treatment control condition and a delayed treatment control condition. As patients with chronic medical conditions, such as asthma, are less likely to receive preventive services such as pap testing and mammography ^94-96^, we have chosen an active treatment control condition that seeks to meet a need of patients with asthma, but will not interfere with our main research questions. In addition, Halpern and colleagues noted that subject recruitment and retention is enhanced in studies in which the control group receives an “active” intervention, rather than a placebo intervention ^97^. The control condition is identical to the control condition currently being used in the study to test the effect of the intervention for hypertension control.

### 4. Recruitment and consent process

Highmark members who meet inclusion criteria, based on claims, will be mailed recruitment letters from Highmark. The letters will include a toll-free number to research our study staff. If there is no response for one month after the first mailed letter, the recruitment letter will be sent again to the non-responders. In order to increase minority recruitment for NIH purposes, we will also send a recruitment flyer and letter to zip codes with high concentrations of minorities. No identifying personal health information will be obtained from patients until they call our study line and would like to hear more about our study. At that time, they will be screened over the phone.

Confirming diagnosis of persistent asthma based on the HEDIS criteria will be screened over the phone. Verbal consent will be administered by PSHMC research staff once the participant has been screened over the phone and is deemed eligible. During this time, the study will be explained in detail, subjects will have the opportunity to ask questions, and the summary explanation of research document will be read and reviewed over the phone. Consent will not be administered to non-English speaking participants, as this is an exclusion criteria.

### 5. Procedures to be followed

Potentially interested participants will call a toll-free number and speak to a study coordinator, who will complete a phone screener. The phone screener includes questions in the following categories: basic demographic information, race and ethnicity, exclusion criteria questions, contact information (e.g., phone number) and social security information, so that we are able to mail compensation for participation in the study. Data from this phone screening process will be collected on paper and parts of this form will be later entered into the management module. During this phone screener, the study coordinator will explain the study, ask questions to determine eligibility and then ask once again if the patient may be interested in participating. If the participant is eligible and interested, the study coordinator will read the verbal consent document over the phone. If the participant is interested, eligible and consents to participate in the study over the phone, the study coordinator will inform the participant that he/she is included in the study and will receive an email within the next day from the study, explaining what will happen next. The coordinator will also inform the participant that upon logging into the application for the first time, they will have to read, review, and electronically agree to a Member Authorization Form by Highmark. They will be able to print out this form for their own records. Those that do not agree will not be allowed to participate. Those that do agree will then be randomized automatically, according to an algorithm written in the management module, to either the asthma or preventive care modules. The study coordinator will enter the specific screening information into the application (all screening fields except for mailing address and SSN) after their phone call with the participant and then check a box representing that the participant has completed the phone consent procedure. The participant will then be asked to use the website once every 30 day cycle and within 14 days of their next scheduled visit with their asthma care provider. At baseline, 6 months, and 12 months, they will be asked to complete specific measures online, as outlined below. During other monthly logins, they will be asked to complete short surveys that will then generate feedback based on their group they were randomized to. Women of child bearing potential will be asked monthly if they are currently pregnant and if they state that they are, they will be withdrawn from the study. At the close of the study, Penn State researchers will be authorized to provide Highmark with the names, DOB’s, gender and dates participants electronically agreed to the Member Authorization Form. Highmark will then provide Penn State researchers with access to claims data for all enrolled participants, for the 2 years prior to study participation and up to 2 years post enrollment. Asthma-related claims of hospitalization, outpatient visits, emergency department visits and medications will be examined.

|  | Phone Screener | Base | Every 30 days | CYCLE 6 | CYCLE 13 | Claims |
| --- | --- | --- | --- | --- | --- | --- |
| Age, gender, race, ethnicity (5) | X |  |  |  |  |  |
| Exclusion (smoking amount, Internet access, asthma history, English fluency)(10) | X |  |  |  |  |  |
| Contact and payment information (13) | X |  |  |  |  |  |
| Demographics, height, weight, QOL (14) |  | X |  |  | X |  |
| Asthma Control Test (5) |  | X |  | X | X |  |
| Asthma Control and Quality of Life (11) |  | X |  | X | X |  |
| Health Conditions (14) |  | X |  |  |  |  |
| Patient Satisfaction Questionnaire (19) |  | X |  |  | X |  |
| Patient Involvement (5) |  | X |  |  | X |  |
| Health Literacy (2) |  | X |  |  | X |  |
| Internet Use (29) |  | X |  |  | X |  |
| Asthma Counseling (5) |  | X |  |  | X |  |
| Newest Vital Sign (8) |  | X |  |  |  |  |
| CC Condition Pre-Use Questions (27) |  | X | X* |  | X |  |
| IC Condition Pre-Use Questions (16) |  | X | X* |  | X |  |
| Emergency department visits |  |  |  |  |  | X |
| Medication use and adherence |  |  |  |  |  | x |
| Outpatient provider visits for asthma |  |  |  |  |  | X |
| Adverse events |  |  |  | X | X |  |
| Satisfaction with Intervention |  |  |  |  | X |  |
| Pregnancy Question | X** | X** | X** | X** | X** |  |

* depending on condition randomized to, each subject will complete either the CC or IC questions

** asked to women of child bearing potential only

### 6. Primary and secondary outcome measures

**Primary Outcome measures:** Asthma control, health care utilization, pulmonary function and asthma-relate quality of life. The main aim of the intervention is to improve asthma control and improve quality of care for asthma, in keeping with the 2007 NAEPP Guidelines ^29^. The NAEPP guidelines state that the goals include “minimal or no chronic symptoms day or night” and “no limitations on activities” ^29^. We hypothesize that each of these measures will be improved in the IC, compared to the CC.

Asthma Control: We will measure asthma control using the Asthma Control Test (ACT), at baseline, 6 months, and 12 months. The ACT asks patients about several aspects of their asthma control over the past 4 weeks: a) whether their asthma interfered with their activities, b) how often they had shortness of breath from asthma, c) how often they used their rescue inhaler, d) how often they had asthma symptoms at night and e) an overall impression of their asthma control. The ACT has a test-retest reliability of 0.84 and compares closely with a specialist’s rating of asthma control, the need for change in patient’s therapy and percent predicted FEV1, future asthma control and future emergency department visits ^98-100^. The ACT is also sensitive to changes in asthma treatment ^99^. The overall agreement between ACT and the specialist’s rating ranged from 71% to 78% depending on the cut points used, and the area under the receiver operating characteristic curve was 0.77 ^98^. An ACT score of ≥ 20 will be used as a cutoff for determining “controlled” v. “uncontrolled” asthma, as this cutoff demonstrated the highest area under the ROC curve. The use of the ACT, as well as the cutoff of 20 for determining control are supported in the 2007 NAEPP Guidelines on page 345 ^26^. We hypothesize that, at 12 months, 70% of subjects in the IC condition will have an ACT of ≥ 20 versus only 55% in the CC condition.

Asthma-Related Emergency Department Visits: Improvements in asthma control can decrease future use asthma-related emergency room visits, so we will measure asthma-related emergency department visits ^6,100,101^. Claims data are valid measures, and superior to other means, such as self-report, for measuring asthma-related emergency department visits. ^102,103^. Highmark will send us claims data, at the end of the study, which will identify asthma-related emergency department claims. This is data that is already being analyzed by Highmark, in ways that are standardized by the National Committee for Quality Assurance (NCQA), for reporting the quality of care delivered to patients with asthma ^7^. In the NCQA reporting, asthma-related emergency department visits are one way to identify patients with persistent asthma ^7^. We will take advantage of that reporting mechanism in the current study.

Asthma Control and Quality of Life: We will use questions about asthma control and quality of life from the National Asthma Survey, from the CDC and published by Halterman and colleagues (Halterman, Aligne et al. 2000). The instrument identifies several aspects of asthma control and how it impacts quality of life, including days with symptoms, days symptom-free, emergency room visits, urgent doctor visits, asthma attacks, severity of asthma attacks and the degree to which asthma impairs function. It is not clear how many of these questions are validated, but they are used as outcome studies in a great number of asthma clinical trials and have strong face validity and national norms is helpful. Drs. Uche Ofoma, Sciamanna and Craig are working on the adult version of the Halterman paper, using the data from the NAS.

**Mediating Variables:** For our purposes, mediating variables are those that the intervention will attempt to modify in order to bring about a change in asthma control and quality of care for asthma. We will examine variables that have been associated with asthma control and quality of care for asthma and those that have been targeted by the intervention and therefore may mediate the effect of the intervention on the above outcome variables. We hypothesize that each of these variables will mediate the effect of the intervention.

Intervention fidelity: Use of interactive website before asthma care provider visits. Use of the interactive website will be tracked passively. Each login by study subjects will be recorded, as will the duration of each login, the number of pages viewed, any data entered and the number of times that subjects received the computer-tailored feedback. The active part of the intervention is felt to be the computer-tailored feedback, so this measure will represent the “dose” of the intervention. Dose will be measured as a proportion of asthma provider visits before which the website was used, expressed as a percentage. The number of visits with an asthma care provider will be obtained from chart review, and will be the denominator for this measure. The number of visits that subjects used the website within two weeks prior to these visits will be the numerator for this measure. We consider visits to an asthma care provider to be any visits to the patients’ primary care provider as well as any visits that the patient had with a pulmonary medicine specialist during the study. Upcoming visit dates will be tracked using brief monthly emailed surveys to patients. These surveys will seek to clarify the date(s) of planned visits to asthma care providers, including primary care providers and specialists. All patients who have not used the website within 14 days before their planned visit to an asthma care provider will be sent an automated email reminder, encouraging subjects to use the site or to use a toll-free number to call the study staff if they are having trouble using the website. These email reminders will be repeated 7 days before their next doctor visit and then 3 days before if they have still not used the website. These methods were successful at ensuring a 90% fidelity rate in a pilot RCT of the website ^25,27^.

Controller to reliever ratio: There are generally two types of medicines for asthma; those used for quick relief of asthma symptoms (“relievers”), and those that are used daily to prevent asthma symptoms (“controllers”). Using a controller medication is the first step in managing persistent asthma, according to the treatment algorithm (page 343) on the 2007 NAEPP Guidelines ^26^, and has served as a main outcome measure in many quality improvement studies ^66-68,104^. By using more controller medicines, therefore, the number of reliever medicines tends to go down ^105^. The ratio of controller/reliever canisters is strongly predictive of future emergency department visits and, as such, has been used as a measure of asthma control and quality of care ^105-107^. As the intervention is designed to help individuals receive a controller medicine and, for those whose asthma is not controlled, often a second controller medicine, the controller to reliever ratio should be sensitive measure of the effects of the intervention. We will use standard ways to express this variable, which will be calculated as the ratio of the number of controller medications (i.e., inhaled corticosteroid) dispensed during the study year divided by the sum of the number of controller medications dispensed plus the number of canisters of inhaled relievers (e.g., short-acting beta-2-agonists) dispensed during the study year. This ratio ranges from 0 (no controller medications) to 1.0 (no reliever medications) ^105-108^. Grymonpre and colleagues observed that the concordance between claims data on medication acquisition and pill counts was between 79% and 88%, which were both highly significant ^109^. This data will be captured in the electronic claims data from Highmark, as none of the medicines are inexpensive, so patients should find it less expensive to use their prescription drug coverage through Highmark than to pay out of pocket. Similar methods are used by the National Committee for Quality Assurance to estimate the quality of asthma care in over 500 health plans nationally ^7^.

Outpatient asthma care provider visits: The intervention will suggest to some patients, based on rules in the 2007 NAEPP Guidelines, to see an asthma care provider sooner than scheduled (page 345) or to see an asthma specialist (page 288) when none had been seen. It is therefore important to capture these measures of treatment effects. To understand the frequency and timing of primary care and specialist visits for asthma, we will review the Highmark claims database at the end of the 12 month study. The names of providers who are seen will be compared to a database of generalist and specialist physicians that we have used in the past, from SK&A, Inc. Asthma care providers will be determined to be of two types: a) any pulmonologist or allergist seen by the patient, b) any primary care provider seen by the patient who prescribed at least one asthma-related medication during the 12-month study. Visits to these providers will be determined by identifying Highmark claims.

Asthma Self-Management Support Counseling: The 2007 NAEPP Guidelines, as well as previous versions, recommend that physicians should counsel their patients about several aspects of self-management, including recognizing early symptoms of asthma, changing their environment to improve asthma control and creating an asthma action plan ^26,29,110^. To measure the delivery of this care, we will use standardized measures from the 2003 National Asthma Study, summarized in the National Healthcare Quality Report ^9,10^. Using this protocol, we will ask specific questions about counseling they received, over the past year, about recognizing early symptoms of asthma, changing their environment to improve asthma control and creating an asthma action plan.

Medication Adherence: The intervention, in some cases, will suggest that patients discuss medication adherence with their provider. This is based on the recommendation (page 336) that “before increasing therapy, however, the clinician should review the patient’s inhaler technique and adherence to therapy” ^26^. We will measure medication adherence to all asthma controller medications, such as inhaled corticosteroids and long-acting beta-2 agonists, using health care claims for asthma-related medications from Highmark. Health care claims have been shown to be a valid and reliable way to assess medication adherence ^109^. Our measure of adherence will be the Medication Possession Ratio (MPR), a commonly used measure of adherence, that is highly correlated with other measures of adherence and predictive of asthma exacerbations ^111-116^. The MPR measures the percentage of doses available to the patient during a specific time period. Dr. Craig has experience, through multiple clinical trials for asthma, in measuring adherence to asthma medications and will oversee analysis of medication adherence data ^117,118^ . Though medication adherence is not a main focus of the intervention, we hypothesize that, by encouraging patients to discuss medication adherence-related issues with their provider, patients in the IC will have higher rates of adherence to their controller medications than subjects in the CC.

**Moderating variables**: For our purposes, moderating variables are those that the intervention will not seek to modify, yet may lead to a differential response to the intervention.

Demographics: Age, gender, race, ethnicity, educational achievement, marital status and household income will be measured, using standardized instruments such as those from the Behavioral Risk Factor Surveillance System ^119,120^. These variables have been shown to be related to asthma prevalence, asthma quality of care and asthma-related quality of life ^121-123^. We will assess these variables at baseline and use the data to assess whether demographic variables, such as household income, moderate the effect of the intervention on inhaled corticosteroid use or asthma control.

Health Literacy: Several studies have identified the link between health literacy and a wide variety of health outcomes ^124-127^. As the intervention to be tested includes written health-oriented messages, the effects of these messages may be dependent on the individuals’ level of health literacy. To measure health literacy, we will use the Short Form Test of Functional Health Literacy in Adults (s-TOFHLA), which is valid and reliable and is shown to be associated with control of chronic illnesses ^124-127^. The abbreviated form of the s-TOFHLA is a 36-item timed reading comprehension test with multiple choice responses, and contains two health passages. The test is scored on a scale from 0 to 36. Though we will make sure that all feedback is written at not more than the 6th grade reading level, we hypothesize that the effect of the intervention will be greater among patients with higher levels of health literacy.

Health-related Internet use: We will measure subjects’ use of the Internet for health-related activities, using a survey instrument from the Pew Internet and American Life Project ^86,87,128,129^. The instrument measures the use of the Internet for a variety of health-related activities (e.g., looking for information about medications). These survey questions will be asked at baseline and at 12 months. As each recommendation in the computer-tailored feedback includes a link to another reputable website with information on the topic, we hypothesize that the effect of the intervention will be greater among patients who have the greatest increases in using the Internet for health-related information.

### 7. Statistics and sample size justification

Sample size considerations focus on the primary hypothesis posited in the Specific Aims section. The major research goal is to determine the efficacy of having patients use the asthma module of the interactive website, for a period of 12 months, for improving asthma control. The primary outcome will be asthma control, based on the Asthma Control Test (ACT), a valid and reliable 5-question self-report measure of asthma control. ^98-100^. We chose to power the study based on asthma control, as it is a powerful predictor of emergency department visits, activity limitations and missing days of work due to asthma ^6,100,130^, and it is a central goal in both Healthy People 2010 as well as in the 2007 NAEPP Guidelines ^26,131^. We chose not to power the study on improving the use of controller medications, such as inhaled corticosteroid use, as the rates of use of these medications has increased greatly over the past decade, which would lead to a ceiling effect ^7^. Despite the broad use of these controller medications, however, asthma control rates are still in the 50% range, so we will not suffer from such a ceiling effect ^5^. Our estimated baseline rate of 55% is based on two studies of asthma control in similar groups of insured patients that both showed asthma control rates in the 50% range ^5,6^. For example, Schatz and colleagues observed that 48.4% of 570 insured adults with asthma had an Asthma Control Test (ACT) score of less than 20, the same measure of asthma control that we are using in the current study ^5^. Vollmer and colleagues reported that 48% of 5,181 insured adults with asthma reported good asthma control, using a slightly different measure of asthma control, the Asthma Quality of Life Questionnaire ^6^. We expect a slightly higher baseline control rate, as patients who volunteer for clinical trials tend to be healthier than those who do not ^132^. This expected effect size is based on a meta-analysis by Stone and colleagues that examined the efficacy of reminders, given to patients, on rates of adult immunization and cancer screening services ^16^. Patient reminders, similar to what we plan to use in the current study, significantly increased immunizations (odds ratio, OR=2.5), mammography utilization (OR=2.3), cervical cytology screening (OR=1.7) and colon cancer screening (OR=2.8). These effect sizes are consistent with reviews by other investigators ^23,50,133^. In the current study, we will be powered to detect an effect size that translates to a relative risk of 1.3 (0.70 /0.55) and odds ratio of 1.9. We chose this conservative effect size to address the fact that we are not examining a process of care but an outcome of care. While we expect the intervention to lead to increases in the number of controller medications used, additional medications do not always help patients. Also, we need to be sensitive to the possible differences in efficacy between interventions to increase adult preventive services (e.g., pap testing) and studies such as ours that seek to change chronic disease management practices. We considered powering on a numerical change in the Asthma Control Test (ACT), our main outcome measure, but chose not to do this as there is less consensus about what represents a meaningful change in the ACT than there is about what represents appropriate asthma control ^5,98^. We will, however, analyze changes in the ACT as a continuous variable, in our secondary analyses. Formulas based on the chi-square test were used to derive sample size calculations from the following assumptions. We assume a type I error rate (alpha) of 5% (0.05), 80% (0.80) power, and a two-tailed test, which allows us to detect significant differences in either direction. We expect a 12-month retention rate of 80%, although we believe that our procedures will substantially minimize the likelihood of attrition. We considered randomizing providers but felt that, given the number of providers in the two geographical areas, it was very unlikely that a physician would have more than one subject enrolled in the study. We will, however, track the name of the asthma care provider, so that we can include this variable in a sensitivity analysis, to be certain that adding this variable does not change the conclusions of the data analyses. In order to achieve 80% power to detect a difference of 70% vs. 55% between intervention and control group, we will need to randomize 408 patients into the two conditions at baseline. This assumes that no more than 20% are lost to follow-up at 12 months, leaving no fewer than 326 subjects available for analysis at the end of the study. We do concede, however, that smaller differences in asthma control may be clinically important, particularly on a population level. To power the study on a 10% difference in asthma control, however, we would have needed to recruit 940 subjects, which we felt would unnecessarily increase the cost and complexity of the study.

Preliminary data analysis plan: All analyses will be conducted using SAS Version 9. Initially, descriptive statistics such as the minimum, maximum, range, median, quartiles, mean, and standard deviation for each continuous variable and frequencies and percentages for each categorical variable will be used to summarize all variables as well as detect outliers, data entry mistakes, and missing values. These descriptives will be used to “clean” the data prior to analysis. Exploratory graphical techniques will be used to further examine these data. The normality of the distribution of mediators of interest, both in their original and transformed state (if necessary), will be examined with a normal probability plot when applicable. Longitudinal plots will be used to identify trends over time that are both population- and individually-based.

Differential dropout and attrition: Initially, the two randomized groups will be compared on important demographic and other baseline variables to ensure successful randomization. If it is determined that the groups, by chance, are significantly different on some measures, we will include those theoretically meaningful confounders as covariates in subsequent analyses. T-tests, ANOVA, and/or chi-square tests of independence comparing those who complete the study with those lost to follow-up will be performed on baseline demographic variables, asthma control, asthma-related quality of life, and baseline use of controller medications to identify potential differential dropout. These comparisons ensure equality of intervention and comparison groups after accounting for study attrition. For the primary outcome, we will apply intention to treat (ITT) principles with all available data included in the first set of analyses ^134^. Second, subjects lost to follow-up will be conservatively assumed to have not changed their level of asthma control between baseline and 12 months. Similar findings between both approaches would increase confidence in the results overall.

Missing data: For exploratory analyses, if the combined rate of missingness on predictor variables is less than 5%, the missing data will not be replaced. In the event of unanticipated higher levels of missing data, we need to examine the nature of the missing values. If it is believed that variations in attrition can be explained by observed variables (e.g. if those who are observed to have poorer asthma control at baseline are more likely to drop out), this corresponds to the missing at random (MAR) assumption and the models as we have specified them will give valid inferences because they are likelihood-based ^135^, and no special adjustments are necessary. If instead we believe that some of the variability in attrition might further be explained by variables not observed, then we will employ multiple imputation ^136^ (i.e., using PROC MI in SAS) which have been shown to yield estimates that are comparable to those that would be obtained if the data were all present. Because such imputations are necessarily based on assumptions that cannot be verified, we will carry out sensitivity analyses to investigate violations of these assumptions.

Analysis of main outcome: Data analysis will focus on the primary hypotheses that significantly more subjects in the IC will have their asthma controlled, an Asthma Control Test (ACT) score of ≥ 20, than subjects in the CC. This dichotomized version of the ACT at 12 months is the primary outcome. We considered powering on a numerical change in the Asthma Control Test (ACT), our main outcome measure, but chose not to do this as there is less consensus about what represents a meaningful change in the ACT than there is about what represents appropriate asthma control ^5,98^. Logistic regression will be used to analyze asthma control at 12 months. Logistic regression allows for inclusion of the two dichotomous strata. Also, it is possible that the groups may have differences at baseline in potential confounders (e.g. socio-economic status, asthma symptoms) that may contribute to group differences in the 12 month event rate between the two conditions. Logistic regression permits inclusion of these potential confounders as covariates. Additionally, as the ACT is measured every 3 months, the logistic regression model can be extended using the generalized estimating equations (GEE) approach of Zeger and Liang (1986) with a logit link so that the repeated measures on each subject can be included in the analyses ^137^. Thus, asthma control can be examined at any of the preceding time points as well. Tests of the primary hypotheses about intervention effects will be formulated directly in terms of the main effect of intervention. We will also assign a create variables for physicians’ practices, so that physicians who practice together will be given the same variable number. In our analyses we first perform intent-to-treat (ITT) analyses and then will perform analysis including physician- and practice-level variables, while accounting for clustering.

Secondary analyses: The secondary hypotheses, taken from the Specific Aims, are that subjects in the IC will have fewer asthma-related emergency department visits, greater gains in their asthma-related quality of life, greater increases in their controller-to-reliever medication ratio, greater satisfaction with asthma treatment and greater adherence to their asthma controller medications, after 12 months, than will patients in the CC. Statistical analyses for these hypotheses will follow the analytic plan as described for the primary outcomes. Secondary outcomes that are dichotomous (e.g. improved asthma-related quality of life) will be fit using the GEE approach with a logit link, which extends the logistic regression model, as described previously. Outcomes that are continuous (e.g. asthma control, adherence) will be analyzed using the linear mixed-effects model developed by Laird and Ware (1982) which extends linear regression to allow for repeated measurements. Ordinal measures will be estimated as a cumulative odds regression model ^138^ after satisfying the assumptions of proportionality between levels of the outcome and the explanatory variables. When this assumption cannot be satisfied, nonproportional models, such as logistic regressions between adjacent categories, will be examined.

Analyses of mediating and moderating processes: The analytic procedures outlined provide a convenient way of identifying effect modifiers and confounders, while modeling the underlying relationship of interest. As noted in the analytic plan for the primary outcome, the analysis of moderators will be undertaken by creating interaction terms between treatment and covariates of interest, such as asthma-related quality of life ^139^, health literacy and socio-demographic characteristics. We will also examine the effects of treatment fidelity and intervention dosage (e.g. number of visits to web site, total time spent on web site) as potential moderators of intervention efficacy. Mediational analyses will be conducted in the form proposed by Baron and Kenny ^140^. Briefly, this entails four steps in which it is demonstrated that there is: a) a significant association between the independent variable and the dependent variable; b) a significant association between the independent variable and the mediators; c) a significant association between the mediator and outcome; and d) a meditational effect is argued to occur when a significant decrease in the relation between the independent (e.g. treatment) and dependent (e.g. asthma control) variables upon the forced entry of the proposed mediators into the model at the final step. We will examine each mediator separately, as joint estimation makes estimation and interpretation of the effect of each potentially mediating variable problematic. Data for these analyses will be obtained from online surveys, date stamps from the interactive website and from the timing of physician visits from chart reviews. For example, we can test whether use of the interactive website influences use of other asthma controller medications (e.g., salmeterol). However, we will also explore various temporal combinations between the exogenous (treatment), endogenous mediators (asthma specialist visits) and outcomes and we will pay particular attention to the temporal ordering of key events and measurement periods.

Ancillary analyses: We plan a detailed tracking and analysis of the delivery and receipt of the intervention components in both the IC and CC conditions. Intervention components will be collected and analyzed using an intervention checklist administered to the subject after the first physician visit, as well as passive monitoring of website usage for all subjects. An implementation index will be calculated for all subjects based on which intervention components each subject was exposed to and for what duration/intensity. This continuous measure will be utilized as a covariate in outcome analyses. Further, we plan to explore the receipt of each treatment component and identify subject characteristics (e.g. age, gender) that are associated with receipt/utilization of a given component. This analytic strategy is particularly useful for gauging utilization of the website. First, we will use descriptive methods to examine total time spent on the website, number of pages viewed and number of times the computer tailored feedback was viewed, all of which can be correlated in relation to the timing of doctor visits, as well as asthma control. Next, we will examine website usage as an outcome variable to characterize which types of patients are more likely to utilize the website. Finally, we plan to examine utilization of the website as predictor of the main study outcomes. These analyses will also be important to provide: a) feedback for website design and management; b) internal validity checks on utilization, which represents dosage and exposure to the intervention; and c) a computation of the effect of dosage, which will be used as a covariate in the prediction of outcomes on the hypothesis that greater utilization of the internet will increase the likelihood of positive outcomes in key intervention indicators, such as use of asthma controller medications. These analyses will permit conclusions regarding the degree to which patient characteristics influence utilization of the website as well as whether the time spent viewing and content of pages viewed influences study outcomes.

**8. Risks and Discomforts**

The main discomforts or risks in this study is loss of confidentiality, psychological stress induced by the questions in the surveys, and receiving incorrect recommendations for asthma care. These risks are rare.

### 9. Benefits

Participants may experience better communication with their primary care providers and improved asthma and preventive care. This project may also provide a new way to foster doctor-patient communication and improve the quality of asthma and preventive care.

### Safeguards and Confidentiality

Confidentiality will be maintained by numerically coding all data, by disguising identifying information and by keeping all data in locked file drawers. All information obtained from subjects will be accessible only to research staff. In addition, the website currently uses the following methods to assure the confidentiality and security of patient data: The website and data collected are stored on PHS servers. Data transmissions over the network will be encrypted using a 128-bit Secure Sockets Layer (SSL) encryption standard, for which PHS has its own security certificate. The SSL protocol provides data security layered between application protocols (such as Hypertext Transfer Protocol (HTTP) and Transfer Control Protocol/Internet Protocol (TCP/IP). This security protocol provides data encryption, server authentication, message integrity, and client authentication for a TCP/IP connection. Computer network security is maintained through network hardware, user authentication, and user roles within the WebAsthma application and its corresponding database. All computers connect through an Internet firewall that prevents unauthorized users from gaining access to the internal network structure. The firewall protects the computer network, and PHS staff members are experienced with the requirements for database access through an active firewall. The local firewall is monitored continuously and equipment logs are reviewed daily for abnormalities or security issues. The database roles facilitate or restrict access to specific website functionality. The database application meets the security requirements of HIPAA.

**Figure 2: Computer Network**

Subjects will be told to skip any items on the questionnaire that they would prefer not to answer, which will reduce the likelihood of psychological stress induced by the questionnaires. Every effort will be made to verify that the feedback given to subjects is accurate. To do this we will 1) work with Dr. Craig to rewrite the feedback to ensure that it is up to date with consensus treatment recommendations, 2) have the feedback reviewed by a primary care provider (Dr. Sciamanna) and 3) perform extensive software testing, given a range of test cases. Also, as the recommendations in the feedback require a physician to implement, the participating primary care physicians will form an additional layer of quality control by implementing recommendations from the reports that they deem to be appropriate for the specific patient.

At the end of the study, Penn State researchers will provide Highmark with a list of participant names, DOB’s, gender, and date they agreed to the member authorization form. This list will be sent through a secure Highmark server and only those we are working in collaboration with on the asthma project will have access to this list in order to pull claims data on these participants. The claims data information will then be sent through a secure server to Penn State in order to minimize the risk of breach of confidentiality.

### 11. Data Safety and Monitoring Plan

### This study involves minimal risk to subjects as they are only interacting with a website to see if it improves their asthma control. Oversight for the conduct of the study will be provided by the Principal Investigator. Adverse events are not anticipated, but any occurring will be documented and reported according to HSPO policies and procedures. The principal investigator will be solely responsible for data collection and verification, and review of cumulative adverse events. Confidentiality will be protected by utilizing a code number as the only identifier for each subject and the master list will be kept under lock and key. Cumulative adverse events and study progress summary will be communicated to the IRB at the time of the continuing review.

###

### 12. Compensation

Participants will be compensated in the form of gift cards for up to a total of $85 over the course of the study. They will receive $25 for successful completion of the baseline survey. For completing the 6 month measure they will receive $10. After the completion of the follow-up measures, they will receive $50.

###

### 13. Drugs, devices or biologics

N/A

***IV. Investigator qualifications and roles in the conduct of the study***

Principle Investigator: Christopher N. Sciamanna, MD, MPH

Sciamanna CN, Nicholson RA, Lofland JH, Manocchia M, Mui S, Hartmann CW. Effects of a website designed to improve the management of migraines. Headache. January 2006;46:92-100.

Blanch DC, Sciamanna CN, Lawless HK, Diaz JA. Effect of the Internet on the Doctor-Patient Relationship: A Review of the Literature. Journal on Information Technology in Healthcare 2005;3(3): 179-201.

Diaz JA, Sciamanna CN, Stamp MJ, Evangelou E, Ferguson T. e-Patients’ Expectations: What Types of Internet Guidance Do e-Patients’ Want from their Physicians? In press, Journal of General Internal Medicine.

Sciamanna CN, Harrold LR, Manocchia M, Walker NJ, Mui S. The effect of web-based, personalized, osteoarthritis quality improvement feedback on patient satisfaction with osteoarthritis care. In press, American Journal of Medical Quality.

Sciamanna CN, Novak SP, Marcus BH, Goldstein MG. Patient attitudes toward using computers to encourage their doctors to counsel about health behaviors: effects of using a computer in a doctor's office. In press, International Journal of Medical Informatics.

Co-Investigator, Timothy Craig, D.O.

Coreale C, Walker C, Craig T. Atopic Dermatitis: A Review of the Literature. The American Family Physician 1999;60:1191-8.

Leon E, Craig T. Antifungals in the treatment of Allergic Bronchopulmonary Aspergillosis. Annals of Allergy, Asthma and Immunology. Annals of Allergy, Asthma and Immunology 1999;82:511-517.

Milan N, Craig T. Drugs of concern for the patient with asthma. JAOA 1998:98;S10;13-18.

Golden S, Lehman M, Mauger E, Teets S, Craig T. The effect of topical Azelastine on the symptoms of rhinitis, sleep and daytime fatigue in perennial allergic rhinitis. Annals of Allergy Asthma and Immunology 2000;85:53-57.

Craig T, Mende C. Frequent allergic and allergic like reactions to medications. Postgraduate Medicine 1999;105:173-181.

Fish J, Craig T, Bensch G, Berger W, Bernstein D, Bronsky E, Harrison J, et al. Inhaled mometasone furoate reduces oral prednisone requirements while improving disease parameters and quality of life in severe persistent asthma. Journal Of Allergy and Clin Immunol 2000;106:852-860.

Berlin J, Craig T. A comparison between azelastine and flunisolide on the effect of sleep, and somnolence in allergic rhinitis. JAOA 2000;100:S8-13.

Co-Investigator: Michele L. Shaffer, PhD

Shaffer ML, Chinchilli VM. A likelihood-based, counterfactual approach to accounting for treatment failures in clinical trials. J Biopharm Stat 2003; 13(3):481-494.

Shaffer ML, Chinchilli VM. Bayesian inference for randomized clinical trials with treatment failures. Stat Med 2004; 23(8):1215-1228.

Watterberg KL, Gerdes JS, Cole CH, Aucott SW, Thilo EH, Mammel MC, Couser RJ, Garland JS, Rozycki HJ, Leach CL, Backstrom C, Shaffer ML. Prophylaxis of early adrenal insufficiency to prevent bronchopulmonary dysplasia: a multicenter trial. Pediatrics 2004; 114(6):1649-1657.

Liu W, Zhao W, Shaffer ML, Icitovic N, Chase GA. Modelling clinical trials in heterogeneous samples. Stat Med 2005; 24(18):2765-2775.

Shaffer ML, Chinchilli VM. Including multiple imputation in a sensitivity analysis for clinical trials with treatment failures. Contemp Clin Trials, accepted June 2006.

## V. Facilities

Web-based asthma management module.

## VI. References

1. Measuring childhood asthma prevalence before and after the 1997 redesign of the National Health Interview Survey--United States. *MMWR Morb Mortal Wkly Rep.* 2000;49(40):908-911.

2. Gendo K, Sullivan SD, Lozano P, Finkelstein JA, Fuhlbrigge A, Weiss KB. Resource costs for asthma-related care among pediatric patients in managed care. *Ann Allergy Asthma Immunol.* Sep 2003;91(3):251-257.

3. Stock S, Redaelli M, Luengen M, Wendland G, Civello D, Lauterbach KW. Asthma: prevalence and cost of illness. *Eur Respir J.* Jan 2005;25(1):47-53.

4. *The State of Health Care Quality.* Washington, D.C.: National Committee for Quality Assurance;2005.

5. Schatz M, Mosen DM, Kosinski M, et al. Predictors of asthma control in a random sample of asthmatic patients. *J Asthma.* May 2007;44(4):341-345.

6. Vollmer WM, Markson LE, O'Connor E, et al. Association of asthma control with health care utilization and quality of life. *Am J Respir Crit Care Med.* Nov 1999;160(5 Pt 1):1647-1652.

7. *The State of Health Care Quality.* Washington: National Committee for Quality Assurance;2006.

8. Ma J, Stafford RS. Quality of US outpatient care: temporal changes and racial/ethnic disparities. *Arch Intern Med.* Jun 27 2005;165(12):1354-1361.

9. *National Healthcare Quality Report.* Rockville: Agency for Healthcare Research and Quality; December 2006.

10. Asthma self-management education among youths and adults--United States, 2003. *MMWR Morb Mortal Wkly Rep.* Sep 7 2007;56(35):912-915.

11. Cabana MD, Rand CS, Powe NR, et al. Why don't physicians follow clinical practice guidelines? A framework for improvement. *Jama.* 1999;282(15):1458-1465.

12. Grant RW, Cagliero E, Dubey AK, et al. Clinical inertia in the management of Type 2 diabetes metabolic risk factors. *Diabet Med.* Feb 2004;21(2):150-155.

13. Phillips LS, Branch WT, Cook CB, et al. Clinical inertia. *Ann Intern Med.* Nov 6 2001;135(9):825-834.

14. Shah BR, Hux JE, Laupacis A, Zinman B, van Walraven C. Clinical inertia in response to inadequate glycemic control: do specialists differ from primary care physicians? *Diabetes Care.* Mar 2005;28(3):600-606.

15. O'Connor PJ. Commentary--improving diabetes care by combating clinical inertia. *Health Serv Res.* Dec 2005;40(6 Pt 1):1854-1861.

16. Stone EG, Morton SC, Hulscher ME, et al. Interventions that increase use of adult immunization and cancer screening services: a meta-analysis. *Ann Intern Med.* 2002;136(9):641-651.

17. Ornstein SM, Garr DR, Jenkins RG, Rust PF, Arnon A. Computer-generated physician and patient reminders. Tools to improve population adherence to selected preventive services. *J Fam Pract.* 1991;32(1):82-90.

18. Turner BJ, Day SC, Borenstein B. A controlled trial to improve delivery of preventive care: physician or patient reminders? *J Gen Intern Med.* 1989;4(5):403-409.

19. Becker DM, Gomez EB, Kaiser DL, Yoshihasi A, Hodge RH. Improving preventive care at a medical clinic: how can the patient help? *Am J Prev Med.* 1989;5(6):353-359.

20. Rosser WW, Hutchison BG, McDowell I, Newell C. Use of reminders to increase compliance with tetanus booster vaccination. *Cmaj.* 1992;146(6):911-917.

21. Kravitz RL, Bell RA, Azari R, Kelly-Reif S, Krupat E, Thom DH. Direct observation of requests for clinical services in office practice: what do patients want and do they get it? *Arch Intern Med.* Jul 28 2003;163(14):1673-1681.

22. Kravitz RL, Epstein RM, Feldman MD, et al. Influence of Patients' Requests for Direct-to-Consumer Advertised Antidepressants: A Randomized Controlled Trial. *Jama.* Apr 27 2005;293(16):1995-2002.

23. Grol R. Improving the quality of medical care: building bridges among professional pride, payer profit, and patient satisfaction. *Jama.* 2001;286(20):2578-2585.

24. Sciamanna CN, Clark MA, Houston TK, Diaz JA. Unmet needs of primary care patients in using the Internet for health-related activities. *J Med Internet Res.* Dec 2002;4(3):E19.

25. Hartmann CW, Sciamanna CN, Blanch DC, et al. A novel website to improve asthma care: Qualitative analysis of end-user experiences. *Journal of Medical Internet Research.* 2007;9(1):e3.

26. *Expert Panel Report 3: Guidelines for the Diagnosis and Management of Asthma.* Bethesda: National Heart, Lung and Blood Institute; August 28 2007.

27. Sciamanna CN, Nicholson RA, Lofland JH, Manocchia M, Mui S, Hartmann CW. Effects of a website designed to improve the management of migraines. *Headache.* January 2006;46:92-100.

28. Sciamanna CN, Harrold LR, Manocchia M, Walker NJ, Mui S. The effect of web-based, personalized, osteoarthritis quality improvement feedback on patient satisfaction with osteoarthritis care. *American Journal of Medical Quality.* 2005;20(3):127-137.

29. *NAEPP Expert Panel Report Guidelines for the Diagnosis and Management of Asthma–Update on Selected Topics 2002*.* Bethesda, MD: National Heart, Lung, and Blood Institute; July 2002.

30. Ford ES, Mannino DM, Homa DM, et al. Self-reported asthma and health-related quality of life: findings from the behavioral risk factor surveillance system. *Chest.* Jan 2003;123(1):119-127.

31. Mularski RA, Asch SM, Shrank WH, et al. The quality of obstructive lung disease care for adults in the United States as measured by adherence to recommended processes. *Chest.* Dec 2006;130(6):1844-1850.

32. Carlton BG, Lucas DO, Ellis EF, Conboy-Ellis K, Shoheiber O, Stempel DA. The status of asthma control and asthma prescribing practices in the United States: results of a large prospective asthma control survey of primary care practices. *J Asthma.* Sep 2005;42(7):529-535.

33. Schonlau M, Mangione-Smith R, Chan KS, et al. Evaluation of a quality improvement collaborative in asthma care: does it improve processes and outcomes of care? *Ann Fam Med.* May-Jun 2005;3(3):200-208.

34. Epping-Jordan JE, Pruitt SD, Bengoa R, Wagner EH. Improving the quality of health care for chronic conditions. *Qual Saf Health Care.* Aug 2004;13(4):299-305.

35. Wagner EH. Meeting the needs of chronically ill people. *Bmj.* 2001;323(7319):945-946.

36. Glasgow RE, Funnell MM, Bonomi AE, Davis C, Beckham V, Wagner EH. Self-management aspects of the improving chronic illness care breakthrough series: implementation with diabetes and heart failure teams. *Ann Behav Med.* 2002;24(2):80-87.

37. Pearson ML, Wu S, Schaefer J, et al. Assessing the implementation of the chronic care model in quality improvement collaboratives. *Health Serv Res.* Aug 2005;40(4):978-996.

38. Greenfield S, Kaplan SH, Ware JE, Jr., Yano EM, Frank HJ. Patients' participation in medical care: effects on blood sugar control and quality of life in diabetes. *J Gen Intern Med.* 1988;3(5):448-457.

39. Huse DM, Roht LH, Alpert JS, Hartz SC. Physicians' knowledge, attitudes, and practice of pharmacologic treatment of hypertension. *Ann Pharmacother.* 2001;35(10):1173-1179.

40. Bazarian JJ, Veenema T, Brayer AF, Lee E. Knowledge of concussion guidelines among practitioners caring for children. *Clin Pediatr (Phila).* 2001;40(4):207-212.

41. Feldman EL, Jaffe A, Galambos N, Robbins A, Kelly RB, Froom J. Clinical practice guidelines on depression: awareness, attitudes, and content knowledge among family physicians in New York. *Arch Fam Med.* 1998;7(1):58-62.

42. Kimura S, Pacala JT. Pressure ulcers in adults: family physicians' knowledge, attitudes, practice preferences, and awareness of AHCPR guidelines. *J Fam Pract.* 1997;44(4):361-368.

43. Cruz-Correa M, Gross CP, Canto MI, et al. The impact of practice guidelines in the management of Barrett esophagus: a national prospective cohort study of physicians. *Arch Intern Med.* 2001;161(21):2588-2595.

44. Cabana MD, Ebel BE, Cooper-Patrick L, Powe NR, Rubin HR, Rand CS. Barriers pediatricians face when using asthma practice guidelines. *Arch Pediatr Adolesc Med.* 2000;154(7):685-693.

45. Cabana MD, Rushton JL, Rush AJ. Implementing practice guidelines for depression: applying a new framework to an old problem. *Gen Hosp Psychiatry.* Jan-Feb 2002;24(1):35-42.

46. Ziemer DC, Miller CD, Rhee MK, et al. Clinical inertia contributes to poor diabetes control in a primary care setting. *Diabetes Educ.* Jul-Aug 2005;31(4):564-571.

47. Berlowitz DR, Ash AS, Glickman M, et al. Developing a quality measure for clinical inertia in diabetes care. *Health Serv Res.* Dec 2005;40(6 Pt 1):1836-1853.

48. Berlowitz DR, Ash AS, Hickey EC, et al. Inadequate management of blood pressure in a hypertensive population. *N Engl J Med.* Dec 31 1998;339(27):1957-1963.

49. Smith WR. Evidence for the effectiveness of techniques To change physician behavior. *Chest.* 2000;118(2 Suppl):8S-17S.

50. Balas EA, Garb CT, Boren SA, Brown GD, Weingarten S, Blumenthal D. Improving preventive care by prompting physicians. *Archives of Internal Medicine.* 2000;160(3):301-308.

51. Balas EA, Austin SM, Mitchell JA, Ewigman BG, Bopp KD, Brown GD. The clinical value of computerized information services. A review of 98 randomized clinical trials. *Arch Fam Med.* 1996;5(5):271-278.

52. Ahluwalia JS, Gibson CA, Kenney RE, Wallace DD, Resnicow K. Smoking status as a vital sign. *J.Gen.Intern.Med.* 1999;14(7):402-408.

53. Demakis JG, Beauchamp C, Cull WL, et al. Improving residents' compliance with standards of ambulatory care: results from the VA Cooperative Study on Computerized Reminders. *JAMA.* 2000;284(11):1411-1416.

54. Rossi RA, Every NR. A computerized intervention to decrease the use of calcium channel blockers in hypertension. *J Gen Intern Med.* 1997;12(11):672-678.

55. Shiffman RN, Freudigman M, Brandt CA, Liaw Y, Navedo DD. A Guideline Implementation System Using Handheld Computers for Office Management of Asthma: Effects on Adherence and Patient Outcomes. *Pediatrics.* 2000;105(4):767-773.

56. Krishna S, Francisco BD, Balas EA, Konig P, Graff GR, Madsen RW. Internet-enabled interactive multimedia asthma education program: a randomized trial. *Pediatrics.* Mar 2003;111(3):503-510.

57. Runge C, Lecheler J, Horn M, Tews JT, Schaefer M. Outcomes of a Web-based patient education program for asthmatic children and adolescents. *Chest.* Mar 2006;129(3):581-593.

58. van der Palen J, Klein JJ, Zielhuis GA, van Herwaarden CL, Seydel ER. Behavioural effect of self-treatment guidelines in a self-management program for adults with asthma. *Patient Educ Couns.* May 2001;43(2):161-169.

59. Perneger TV, Sudre P, Muntner P, et al. Effect of patient education on self-management skills and health status in patients with asthma: a randomized trial. *Am J Med.* 2002;113(1):7-14.

60. Smeele IJ, Grol RP, van Schayck CP, van den Bosch WJ, van den Hoogen HJ, Muris JW. Can small group education and peer review improve care for patients with asthma/chronic obstructive pulmonary disease? *Qual Health Care.* Jun 1999;8(2):92-98.

61. Neri M, Migliori GB, Spanevello A, et al. Economic analysis of two structured treatment and teaching programs on asthma. *Allergy.* May 1996;51(5):313-319.

62. Finkelstein JA, Lozano P, Fuhlbrigge AL, et al. Practice-level effects of interventions to improve asthma care in primary care settings: the Pediatric Asthma Care Patient Outcomes Research Team. *Health Serv Res.* Dec 2005;40(6 Pt 1):1737-1757.

63. Kotses H, Bernstein IL, Bernstein DI, et al. A self-management program for adult asthma. Part I: Development and evaluation. *J Allergy Clin Immunol.* Feb 1995;95(2):529-540.

64. Lozano P, Finkelstein JA, Carey VJ, et al. A multisite randomized trial of the effects of physician education and organizational change in chronic-asthma care: health outcomes of the Pediatric Asthma Care Patient Outcomes Research Team II Study. *Arch Pediatr Adolesc Med.* Sep 2004;158(9):875-883.

65. Davis RS, Bukstein DA, Luskin AT, Kailin JA, Goodenow G. Changing physician prescribing patterns through problem-based learning: an interactive, teleconference case-based education program and review of problem-based learning. *Ann Allergy Asthma Immunol.* Sep 2004;93(3):237-242.

66. Sondergaard J, Andersen M, Vach K, Kragstrup J, Maclure M, Gram LF. Detailed postal feedback about prescribing to asthma patients combined with a guideline statement showed no impact: a randomised controlled trial. *Eur J Clin Pharmacol.* May 2002;58(2):127-132.

67. Feder G, Griffiths C, Highton C, Eldridge S, Spence M, Southgate L. Do clinical guidelines introduced with practice based education improve care of asthmatic and diabetic patients? A randomised controlled trial in general practices in east London. *Bmj.* Dec 2 1995;311(7018):1473-1478.

68. Veninga CC, Lagerlov P, Wahlstrom R, et al. Evaluating an educational intervention to improve the treatment of asthma in four European countries. Drug Education Project Group. *Am J Respir Crit Care Med.* Oct 1999;160(4):1254-1262.

69. Ruoff G, Gray LS. Using a flow sheet to improve performance in treatment of elderly patients with type 2 diabetes. *Fam Med.* 1999;31(5):331-336.

70. Eccles M, Hawthorne G, Whitty P, et al. A randomised controlled trial of a patient based Diabetes recall and Management system: the DREAM trial: A study protocol [ISRCTN32042030]. *BMC Health Serv Res.* 2002;2(1):5.

71. Casebeer L, Roesener GH. Patient informatics: using a computerized system to monitor patient compliance in the treatment of hypertension. *Medinfo.* 1995;8(Pt 2):1500-1502.

72. Tierney WM, Overhage JM, Murray MD, et al. Can computer-generated evidence-based care suggestions enhance evidence-based management of asthma and chronic obstructive pulmonary disease? A randomized, controlled trial. *Health Serv Res.* Apr 2005;40(2):477-497.

73. Lobach DF, Hammond WE. Development and evaluation of a Computer-Assisted Management Protocol (CAMP): improved compliance with care guidelines for diabetes mellitus. *Proc Annu Symp Comput Appl Med Care.* 1994:787-791.

74. Lobach DF. A model for adapting clinical guidelines for electronic implementation in primary care. *Proc Annu Symp Comput Appl Med Care.* 1995:581-585.

75. Lobach DF, Hammond WE. Computerized decision support based on a clinical practice guideline improves compliance with care standards. *Am J Med.* 1997;102(1):89-98.

76. McPhee SJ, Bird JA, Fordham D, Rodnick JE, Osborn EH. Promoting cancer prevention activities by primary care physicians. Results of a randomized, controlled trial. *JAMA.* Jul 24-31 1991;266(4):538-544.

77. McCowan C, Neville RG, Ricketts IW, Warner FC, Hoskins G, Thomas GE. Lessons from a randomized controlled trial designed to evaluate computer decision support software to improve the management of asthma. *Med Inform Internet Med.* Jul-Sep 2001;26(3):191-201.

78. Tierney WM, Overhage JM, Murray MD, et al. Effects of computerized guidelines for managing heart disease in primary care. *J Gen Intern Med.* Dec 2003;18(12):967-976.

79. Porter SC, Forbes P, Feldman HA, Goldmann DA. Impact of patient-centered decision support on quality of asthma care in the emergency department. *Pediatrics.* Jan 2006;117(1):e33-42.

80. Cherry DK, Woodwell DA. National Ambulatory Medical Care Survey: 2005 summary. *Advance Data.* June 2007;387(June 29):1-39.

81. Chaudhry B, Wang J, Wu S, et al. Systematic review: impact of health information technology on quality, efficiency, and costs of medical care. *Ann Intern Med.* May 16 2006;144(10):742-752.

82. Madden M. *Internet Penetration and Impact, April 2006.* Washington, DC: Pew Internet and American Life Project; April 2006.

83. *Three out of four Americans have access to the Internet, according to Nielsen/NetRatings.* New York: NetRatings, Inc.; March 18 2004.

84. Fox S. *Health Information Online: Eight in ten internet users have looked for health information online, with increased interest in diet, fitness, drugs, health insurance, experimental treatments, and particular doctors and hospitals.* Washington: Pew Internet and American Life Project;2005.

85. Boase J, Horrigan JB, Wellman B, Raine L. *The Strength of Internet Ties.* Washington, D.C.: Pew Internet and American Life Project;2006.

86. Raine L, Fox S. *The Online Health Care Revolution: How the Web helps Americans take better care of themselves.* Washington, D.C.: Pew Internet and American Life Project; November 26 2000.

87. Raine L. *Pew Internet Project: Tracking Report.* Washington, DC: The Pew Internet & American Life Project;2002.

88. Fox S, Fallows D. *Internet Health Resources: Health searches and email have become more commonplace, but there is room for improvement in searches and overall Internet access.* Washington, DC: Pew Internet and American Life Project; 16 July 2003.

89. Dvorak JC. Lindows and the $199 Computer. *PC Magazine*2002.

90. Sciamanna CN, Gifford DR, Smith RJ. Design and acceptability of patient-oriented computerized diabetes care reminders for use at the point of care. *Medical Informatics and the Internet in Medicine.* 2004;29(2):157-168.

91. Flay BR, Graumlich S, Segawa E, Burns JL, Holliday MY. Effects of 2 prevention programs on high-risk behaviors among African American youth: a randomized trial. *Arch Pediatr Adolesc Med.* Apr 2004;158(4):377-384.

92. Nelson ME, Layne JE, Bernstein MJ, et al. The effects of multidimensional home-based exercise on functional performance in elderly people. *J Gerontol A Biol Sci Med Sci.* Feb 2004;59(2):154-160.

93. Broderick JE, Stone AA, Smyth JM, Kaell AT. The feasibility and effectiveness of an expressive writing intervention for rheumatoid arthritis via home-based videotaped instructions. *Ann Behav Med.* Feb 2004;27(1):50-59.

94. Fontana SA, Baumann LC, Helberg C, Love RR. The delivery of preventive services in primary care practices according to chronic disease status. *Am J Public Health.* 1997;87(7):1190-1196.

95. Beckman TJ, Cuddihy RM, Scheitel SM, Naessens JM, Killian JM, Pankratz VS. Screening mammogram utilization in women with diabetes. *Diabetes Care.* Dec 2001;24(12):2049-2053.

96. Bell RA, Shelton BJ, Paskett ED. Colorectal cancer screening in North Carolina: associations with diabetes mellitus and demographic and health characteristics. *Prev Med.* Feb 2001;32(2):163-167.

97. Halpern SD, Ubel PA, Berlin JA, Townsend RR, Asch DA. Physicians' Preferences for Active-controlled versus Placebo-controlled Trials of New Antihypertensive Drugs. *J Gen Intern Med.* 2002;17(9):689-695.

98. Nathan RA, Sorkness CA, Kosinski M, et al. Development of the asthma control test: a survey for assessing asthma control. *J Allergy Clin Immunol.* Jan 2004;113(1):59-65.

99. Schatz M, Sorkness CA, Li JT, et al. Asthma Control Test: reliability, validity, and responsiveness in patients not previously followed by asthma specialists. *J Allergy Clin Immunol.* Mar 2006;117(3):549-556.

100. Schatz M, Zeiger RS, Drane A, et al. Reliability and predictive validity of the Asthma Control Test administered by telephone calls using speech recognition technology. *J Allergy Clin Immunol.* Feb 2007;119(2):336-343.

101. Schatz M, Rodriguez E, Falkoff R, Zeiger RS. The relationship of frequency of follow-up visits to asthma outcomes in patients with moderate persistent asthma. *J Asthma.* Mar 2003;40(1):49-53.

102. Dendukuri N, McCusker J, Bellavance F, et al. Comparing the validity of different sources of information on emergency department visits: a latent class analysis. *Med Care.* Mar 2005;43(3):266-275.

103. Reeves MJ, Lyon-Callo S, Brown MD, Rosenman K, Wasilevich E, Williams SG. Using billing data to describe patterns in asthma-related emergency department visits in children. *Pediatrics.* Apr 2006;117(4 Pt 2):S106-117.

104. Premaratne UN, Sterne JA, Marks GB, Webb JR, Azima H, Burney PG. Clustered randomised trial of an intervention to improve the management of asthma: Greenwich asthma study. *Bmj.* May 8 1999;318(7193):1251-1255.

105. Fuhlbrigge A, Carey VJ, Adams RJ, et al. Evaluation of asthma prescription measures and health system performance based on emergency department utilization. *Med Care.* May 2004;42(5):465-471.

106. Lozano P, Finkelstein JA, Hecht J, Shulruff R, Weiss KB. Asthma medication use and disease burden in children in a primary care population. *Arch Pediatr Adolesc Med.* Jan 2003;157(1):81-88.

107. Stafford RS, Ma J, Finkelstein SN, Haver K, Cockburn I. National trends in asthma visits and asthma pharmacotherapy, 1978-2002. *J Allergy Clin Immunol.* Apr 2003;111(4):729-735.

108. Schatz M, Zeiger RS, Vollmer WM, et al. The controller-to-total asthma medication ratio is associated with patient-centered as well as utilization outcomes. *Chest.* Jul 2006;130(1):43-50.

109. Grymonpre R, Cheang M, Fraser M, Metge C, Sitar DS. Validity of a prescription claims database to estimate medication adherence in older persons. *Med Care.* May 2006;44(5):471-477.

110. *Guidelines for the Diagnosis and Management and Asthma.* Bethesda, MD: National Heart, Lung, and Blood Institute;1997.

111. Balkrishnan R, Rajagopalan R, Camacho FT, Huston SA, Murray FT, Anderson RT. Predictors of medication adherence and associated health care costs in an older population with type 2 diabetes mellitus: a longitudinal cohort study. *Clin Ther.* Nov 2003;25(11):2958-2971.

112. Siegel D, Lopez J, Meier J. Antihypertensive medication adherence in the Department of Veterans Affairs. *Am J Med.* Jan 2007;120(1):26-32.

113. Finley PR, Rens HR, Pont JT, et al. Impact of a collaborative pharmacy practice model on the treatment of depression in primary care. *Am J Health Syst Pharm.* Aug 15 2002;59(16):1518-1526.

114. Hess LM, Raebel MA, Conner DA, Malone DC. Measurement of adherence in pharmacy administrative databases: a proposal for standard definitions and preferred measures. *Ann Pharmacother.* Jul-Aug 2006;40(7-8):1280-1288.

115. Andrade SE, Kahler KH, Frech F, Chan KA. Methods for evaluation of medication adherence and persistence using automated databases. *Pharmacoepidemiol Drug Saf.* Aug 2006;15(8):565-574; discussion 575-567.

116. Elliott WJ, Plauschinat CA, Skrepnek GH, Gause D. Persistence, adherence, and risk of discontinuation associated with commonly prescribed antihypertensive drug monotherapies. *J Am Board Fam Med.* Jan-Feb 2007;20(1):72-80.

117. Lazarus SC, Chinchilli VM, Rollings NJ, et al. Smoking affects response to inhaled corticosteroids or leukotriene receptor antagonists in asthma. *Am J Respir Crit Care Med.* Apr 15 2007;175(8):783-790.

118. Inglis CM, Craig TJ. Assessment of care and disease management of patients with asthma in an overseas U.S. Army health clinic. *Mil Med.* Jul 2004;169(7):558-561.

119. Kim C, Beckles GL. Cardiovascular disease risk reduction in the Behavioral Risk Factor Surveillance System. *Am J Prev Med.* Jul 2004;27(1):1-7.

120. Greenlund KJ, Denny CH, Mokdad AH, Watkins N, Croft JB, Mensah GA. Using behavioral risk factor surveillance data for heart disease and stroke prevention programs. *Am J Prev Med.* Dec 2005;29(5 Suppl 1):81-87.

121. Halm EA, Wisnivesky JP, Leventhal H. Quality and access to care among a cohort of inner-city adults with asthma: who gets guideline concordant care? *Chest.* Oct 2005;128(4):1943-1950.

122. Apter AJ, Boston RC, George M, et al. Modifiable barriers to adherence to inhaled steroids among adults with asthma: it's not just black and white. *J Allergy Clin Immunol.* Jun 2003;111(6):1219-1226.

123. Adams RJ, Fuhlbrigge A, Guilbert T, Lozano P, Martinez F. Inadequate use of asthma medication in the United States: results of the asthma in America national population survey. *J Allergy Clin Immunol.* Jul 2002;110(1):58-64.

124. Schillinger D, Grumbach K, Piette J, et al. Association of health literacy with diabetes outcomes. *Jama.* 2002;288(4):475-482.

125. Roter DL, Rudd RE, Comings J. Patient literacy. A barrier to quality of care. *J Gen Intern Med.* 1998;13(12):850-851.

126. Parker RM, Baker DW, Williams MV, Nurss JR. The test of functional health literacy in adults: a new instrument for measuring patients' literacy skills. *J Gen Intern Med.* 1995;10(10):537-541.

127. Baker DW, Parker RM, Williams MV, Clark WS. Health literacy and the risk of hospital admission [see comments]. *Journal of General Internal Medicine.* Dec 1998;13(12):791-798.

128. Raine L, Fox S. *Vital decisions: How Internet users decide what information to trust when they or their loved ones are sick.* Washington, DC: Pew Internet & American Life Project; May 22 2002.

129. Fox S. *Online Health Search 2006.* Washington, D.C.: Pew Internet & American Life Project; October 29, 2006 2006.

130. *Healthy People 2010.* Washington, DC: Centers for Disease Control;2000.

131. *Healthy People 2010.* Atlanta: Centers for Disese Control and Prevention; 1999.

132. Pinsky PF, Miller A, Kramer BS, et al. Evidence of a healthy volunteer effect in the prostate, lung, colorectal, and ovarian cancer screening trial. *Am J Epidemiol.* Apr 15 2007;165(8):874-881.

133. Hunt DL, Haynes RB, Hanna SE, Smith K. Effects of computer-based clinical decision support systems on physician performance and patient outcomes: a systematic review [see comments]. *Jama.* 1998;280(15):1339-1346.

134. Little R, Yau L. Intent-to-treat analysis for longitudinal studies with drop-outs. *Biometrics.* Dec 1996;52(4):1324-1333.

135. Little RJ, Rubin. *Statistical Analysis with Missing Data.* New York: Wiley & Sons; 1987.

136. Rubin DB, Schenker N. Multiple imputation in health-care databases: an overview and some applications. *Stat Med.* Apr 1991;10(4):585-598.

137. Zeger SL, Liang KY. Longitudinal data analysis for discrete and continuous outcomes. *Biometrics.* Mar 1986;42(1):121-130.

138. Agresti A. *Categorical Data Analysis.* Second ed. New York: Wiley; 2002.

139. Juniper EF, Buist AS, Cox FM, Ferrie PJ, King DR. Validation of a standardized version of the Asthma Quality of Life Questionnaire. *Chest.* 1999;115(5):1265-1270.

140. Baron RM, Kenny DA. The moderator-mediator variable distinction in social psychological research: conceptual, strategic, and statistical considerations. *J Pers Soc Psychol.* 1986;51(6):1173-1182.
