# Supplementary material for: Impact of online patient reminders to improve asthma care: A randomized controlled trial
Source: PLoS One. 2017 Feb 3;12(2):e0170447. doi: 10.1371/journal.pone.0170447 (PMC5291361; doi:10.1371/journal.pone.0170447)
Supplement: S1 Table — (DOCX) [file pone.0170447.s003.docx]

**S1 Table. Secondary Outcomes by Asthma Severity.**

| **National Asthma Survey (NAS)** | | | | | | | | | | |
| --- | --- | --- | --- | --- | --- | --- | --- | --- | --- | --- |
| **Outcome** † |  | **Intervention** | | | | **Control** | | | | **P-value** |
|  | **Asthma Severity** | **N** | **Baseline** | **12 Months** | **Change from Baseline** | **N** | **Baseline** | **12 Months** | **Change from Baseline** |  |
|  |  |  |  |  |  |  |  |  |  |  |
| In the past 30 days, on how many days have you had symptoms of asthma? (≤8, %)† | Uncontrolled | 26 | 13.5 | 57.7 | 44.2 | 25 | 2.9 | 44.0 | 41.1 | 0.347 |
|  | Not well controlled | 49 | 29.7 | 69.4 | 39.7 | 53 | 45.2 | 69.8 | 24.6 | 0.242 |
|  | Well controlled | 82 | 90.0 | 87.8 | -2.2 | 90 | 84.3 | 84.4 | 0.1 | 0.758 |
| During the past 30 days, on how many days did symptoms of asthma make you limit your activity? (0, %)† | Uncontrolled | 26 | 2.7 | 34.6 | 31.9 | 25 | 14.7 | 16.0 | 1.3 | N/A |
|  | Not well controlled | 49 | 34.4 | 57.1 | 22.7 | 53 | 17.7 | 60.4 | 42.7 | 0.049 |
|  | Well controlled | 82 | 75.0 | 81.7 | 6.7 | 90 | 62.0 | 73.3 | 11.3 | 0.780 |
| † %, P-value testing for difference in change from baseline between Intervention and Control groups from Binary Generalized Estimating Equations (GEE) model adjusted for denial of insurance | | | | | | | | | | |

| **Use of and Adherence to Asthma Medications** | | | | | | | | | | |
| --- | --- | --- | --- | --- | --- | --- | --- | --- | --- | --- |
| **Outcome** |  | **Intervention** | | | | **Control** | | | | **P-value** |
|  | **Asthma Severity** | **N** | **Baseline** | **12 Months** | **Change from Baseline** | **N** | **Baseline** | **12 Months** | **Change from Baseline** |  |
| Number of Asthma Medications, Medications* | Uncontrolled | 27 | 2.79  (2.05, 3.54) | 2.82  (2.09, 3.56) | 0.03  (-0.51, 0.58) | 25 | 2.11  (1.43, 2.79) | 2.16  (1.49, 2.82) | 0.04  (-0.52, 0.61) | 0.979 |
|  | Not well controlled | 49 | 1.73  (1.09, 2.37) | 2.37  (1.74, 3.01) | 0.64  (0.40, 0.88) | 53 | 1.98  (1.35, 2.60) | 2.19  (1.58, 2.81) | 0.22  (-0.02, 0.45) | 0.014 |
|  | Well controlled | 82 | 1.10  (0.66, 1.55) | 1.53  (1.07, 1.98) | 0.42  (0.22, 0.63) | 90 | 1.18  (0.77, 1.59) | 1.50  (1.09, 1.92) | 0.33  (0.13, 0.52) | 0.494 |
| Number of Asthma Controller Medications* | Uncontrolled | 27 | 1.84  (1.26, 2.43) | 1.79  (1.22, 2.37) | -0.05  (-0.41, 0.31) | 25 | 1.22 (0.69, 1.75) | 1.18  (0.66, 1.70) | -0.04  (-0.41, 0.33) | 0.973 |
|  | Not well controlled | 49 | 1.07  (0.54, 1.60) | 1.42  (0.89, 1.96) | 0.35  (0.17, 0.53) | 53 | 1.15 (0.63, 1.66) | 1.27  (0.75, 1.78) | 0.12  (-0.05, 0.29) | 0.070 |
|  | Well controlled | 82 | 0.86  (0.52, 1.21) | 1.02  (0.67, 1.38) | 0.16  (0.01, 0.32) | 90 | 0.84  (0.52, 1.16) | 1.01  (0.69, 1.34) | 0.17  (0.03, 0.32) | 0.916 |
| Number of Emergency Room Visits* | Uncontrolled | 27 | 0.51  (-0.61, 1.63) | 0.11  (-0.79, 1.01) | -0.40  (-1.09, 0.30) | 25 | 0.08  (-1.04, 1.20) | 0.34  (-0.57, 1.26) | 0.26  (-0.58, 1.10) | 0.231 |
|  | Not well controlled | 49 | 0.28  (-0.07, 0.63) | 0.05  (-0.15, 0.25) | -0.23  (-0.55, 0.09) | 53 | 0.40  (0.07, 0.73) | 0.10  (-0.07, 0.28) | -0.30  (-0.60, 0.01) | 0.765 |
|  | Well controlled | 82 | 0.19  (0.0, 0.39) | -0.01  (-0.13, 0.10) | -0.21  (-0.37, -0.04) | 90 | 0.11  (-0.08, 0.29) | 0.02  (-0.08, 0.13) | -0.08  (-0.25, 0.08) | 0.305 |
| Number of Outpatient Visits* | Uncontrolled | 27 | 3.31  (1.97, 4.65) | 3.75  (2.39, 5.11) | 0.44  (-0.74, 1.62) | 25 | 3.26  (1.92, 4.60) | 3.91  (2.45, 5.38) | 0.66  (-0.77, 2.09) | 0.814 |
|  | Not well controlled | 49 | 3.23  (1.71, 4.75) | 3.04  (1.47, 4.61) | -0.19  (-1.07, 0.69) | 53 | 2.84  (1.44, 4.23) | 2.62  (1.19, 4.05) | -0.22  (-1.05, 0.61) | 0.959 |
|  | Well controlled | 82 | 2.71  (1.83, 3.59) | 2.34  (1.45, 3.24) | -0.36  (-0.81, 0.08) | 90 | 2.41  (1.61, 3.21) | 2.37  (1.57, 3.18) | -0.03  (-0.45, 0.38) | 0.281 |
| * Mean (95% CI), P-value testing for difference in change from baseline between Intervention and Control groups from Linear Mixed Effects Model adjusted for denial of insurance | | | | | | | | | | |
