# Supplementary material for: Impact of online patient reminders to improve asthma care: A randomized controlled trial
Source: PLoS One. 2017 Feb 3;12(2):e0170447. doi: 10.1371/journal.pone.0170447 (PMC5291361; doi:10.1371/journal.pone.0170447)

S1 Appendix. Histograms and Box Plots for Each Continuous Outcome.

Distribution by treatment group for change from baseline to 12 months
Asthma Control Test Score

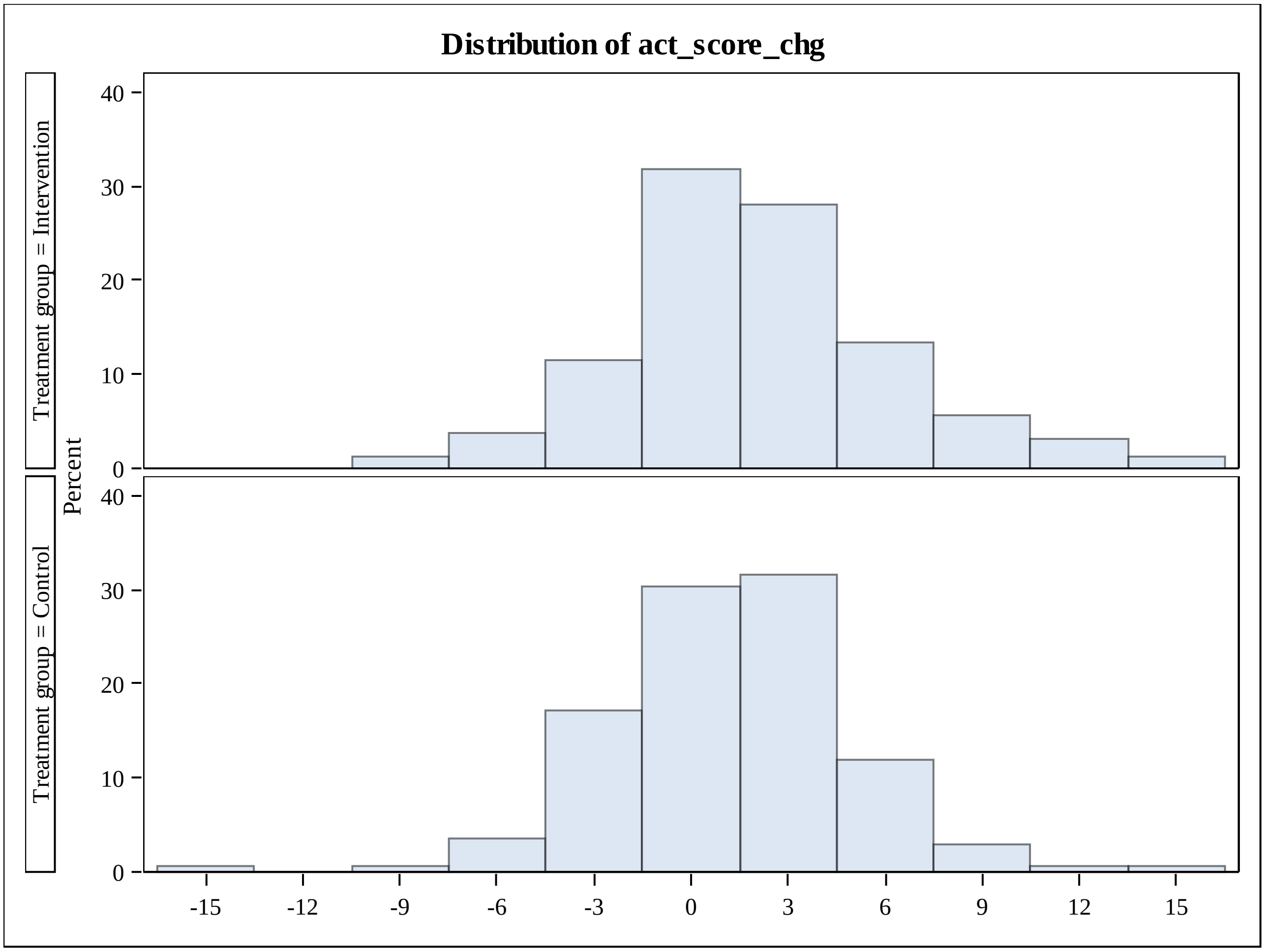

Distribution by treatment group for change from baseline to 12 months
Asthma Control Test Score

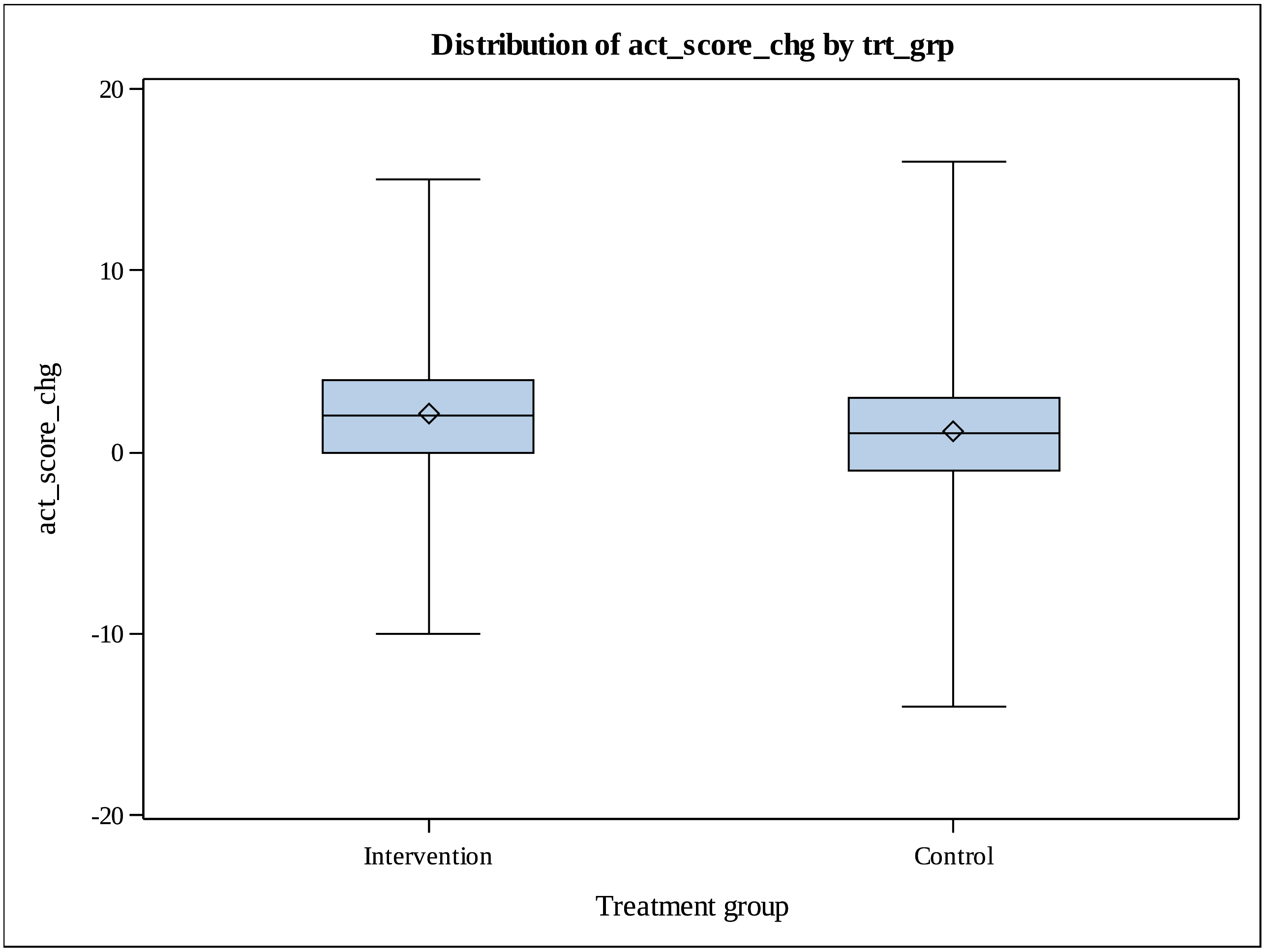

Distribution by treatment group for change from baseline to 12 months
Number of Asthma Medications

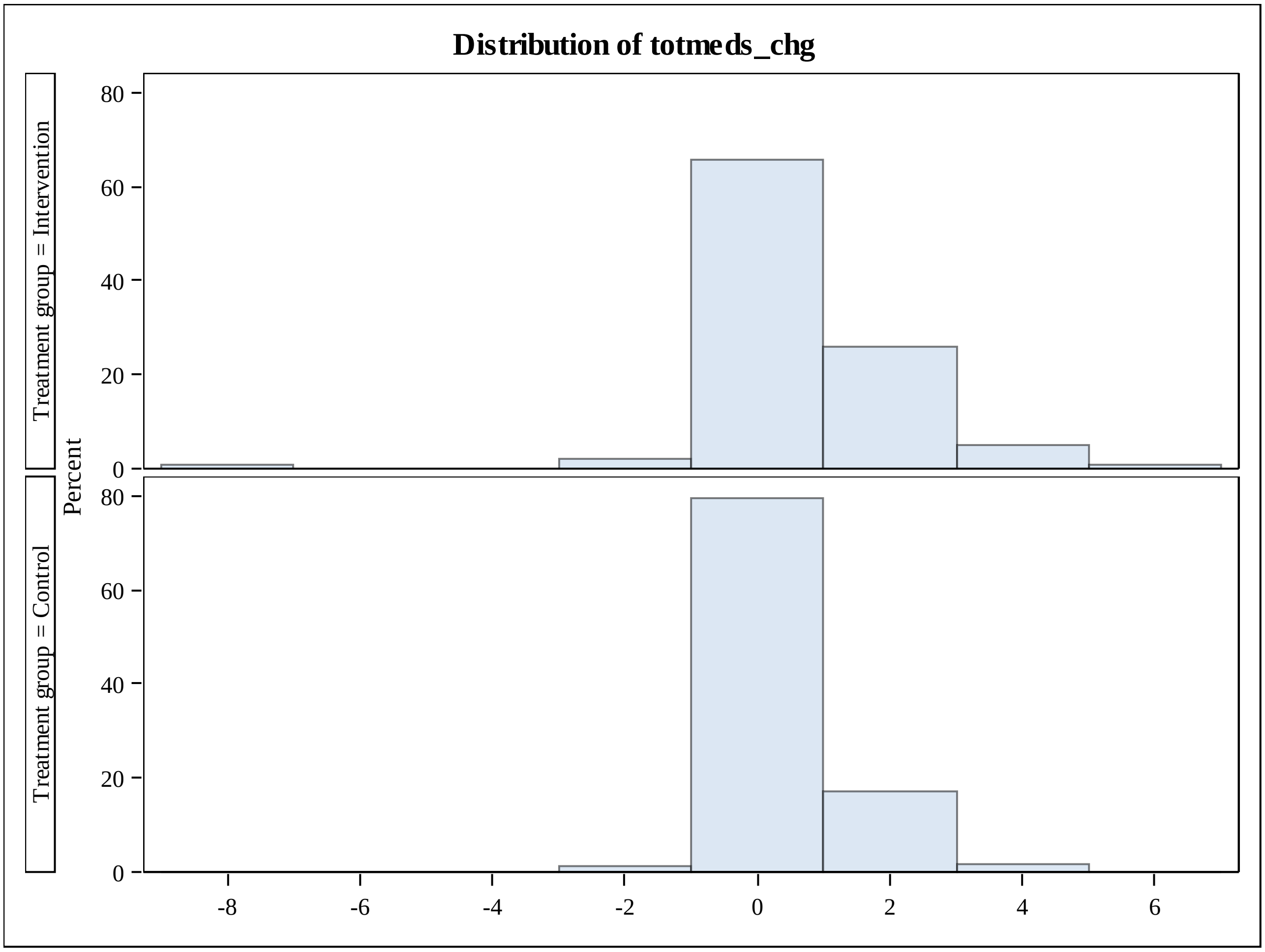

Distribution by treatment group for change from baseline to 12 months
Number of Asthma Medications

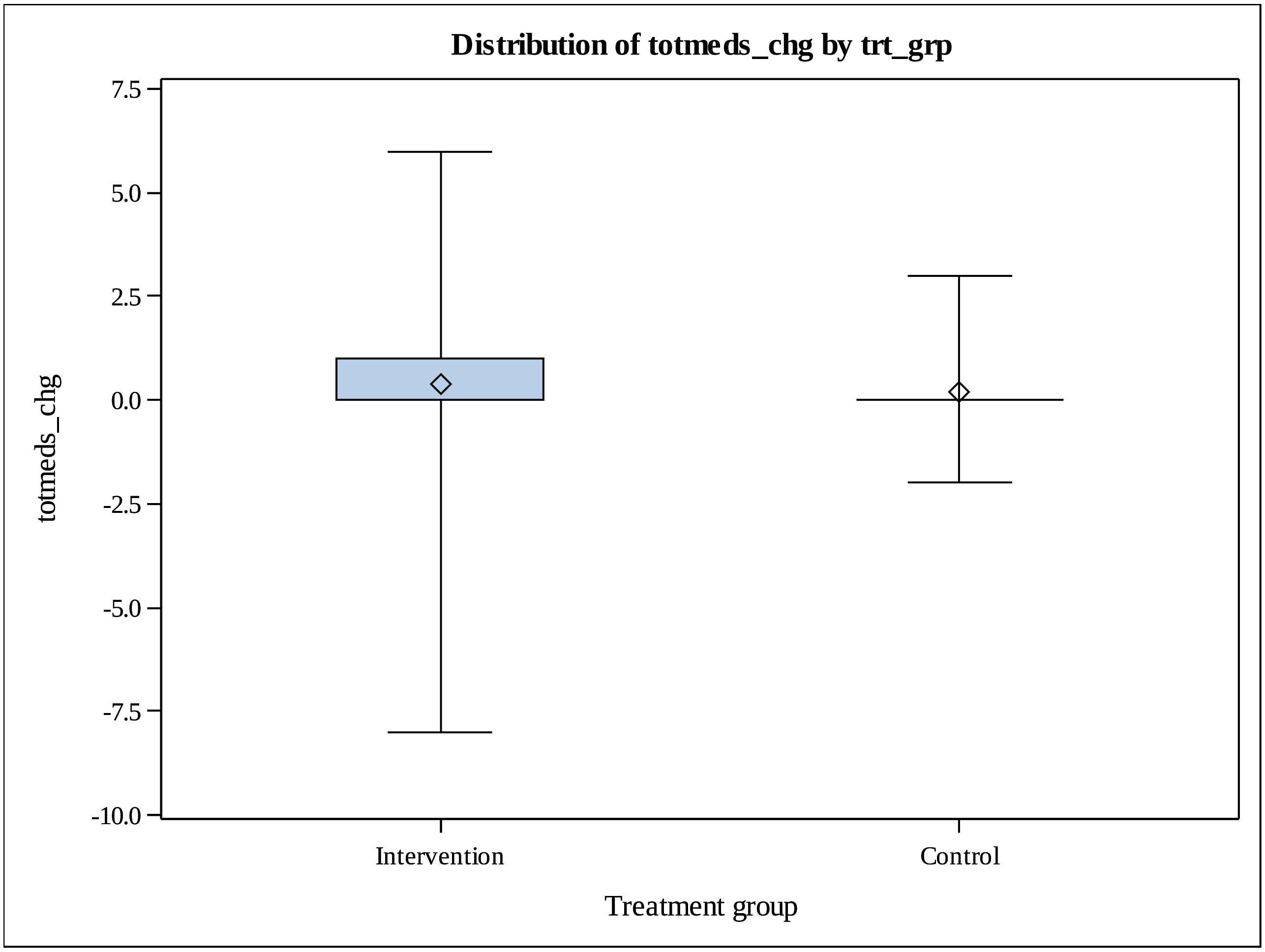

Distribution by treatment group for change from baseline to 12 months
Number of Asthma Controller Medications

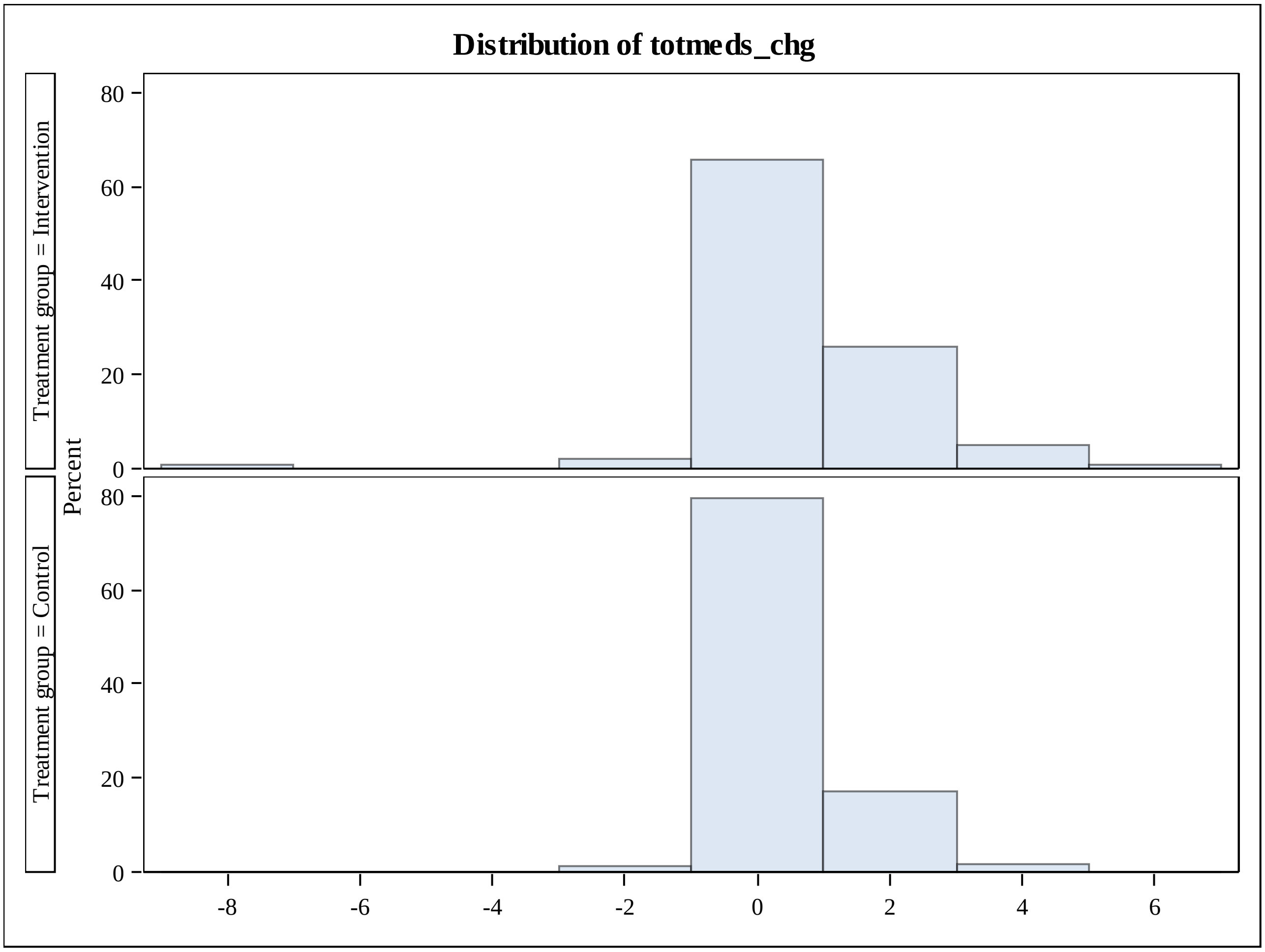

Distribution by treatment group for change from baseline to 12 months
Number of Asthma Controller Medications

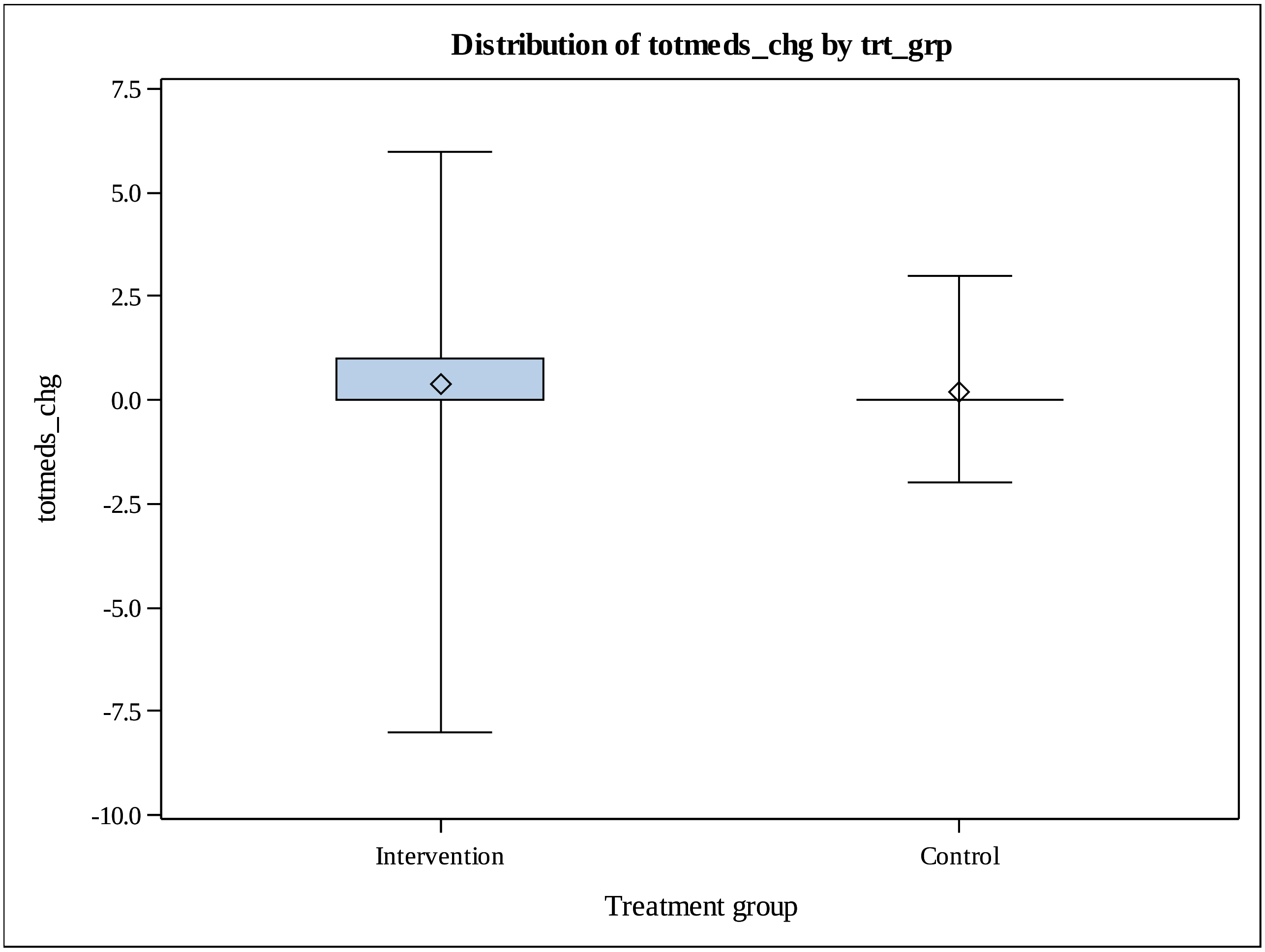

Distribution by treatment group for change from baseline to 12 months
Number of Emergency Room Visits

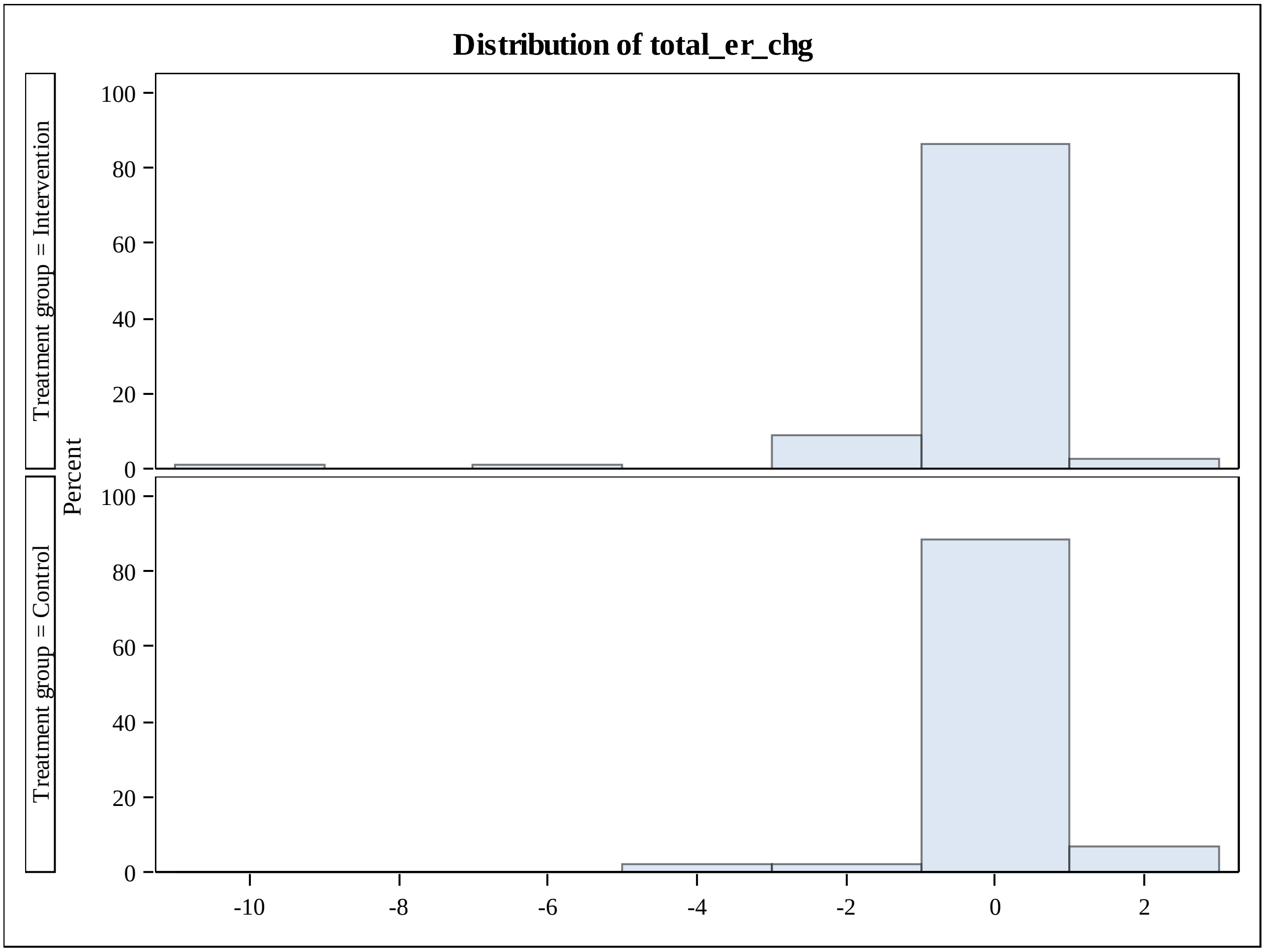

Distribution by treatment group for change from baseline to 12 months
Number of Emergency Room Visits

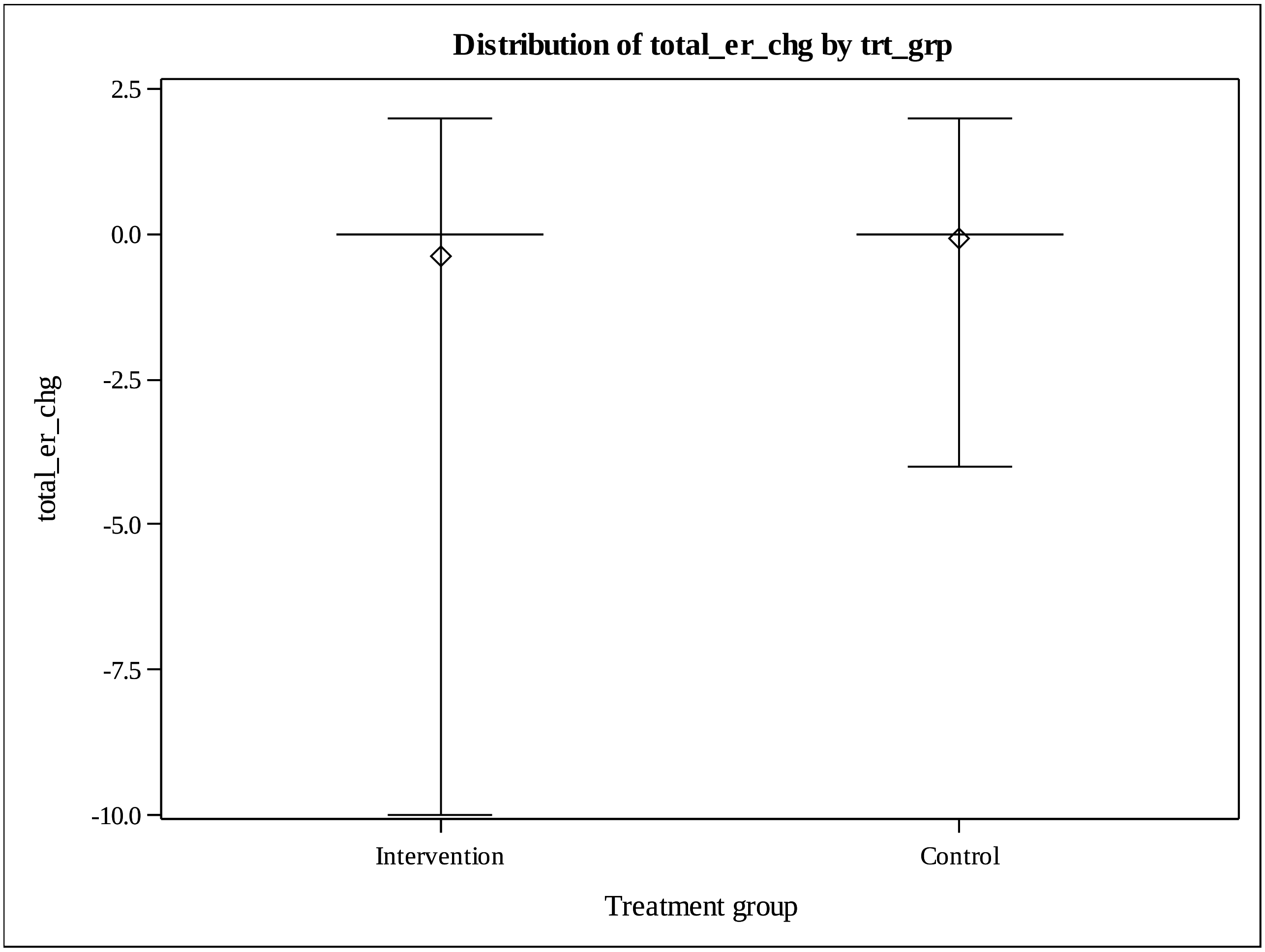

Distribution by treatment group for change from baseline to 12 months
Number of Outpatient Visits

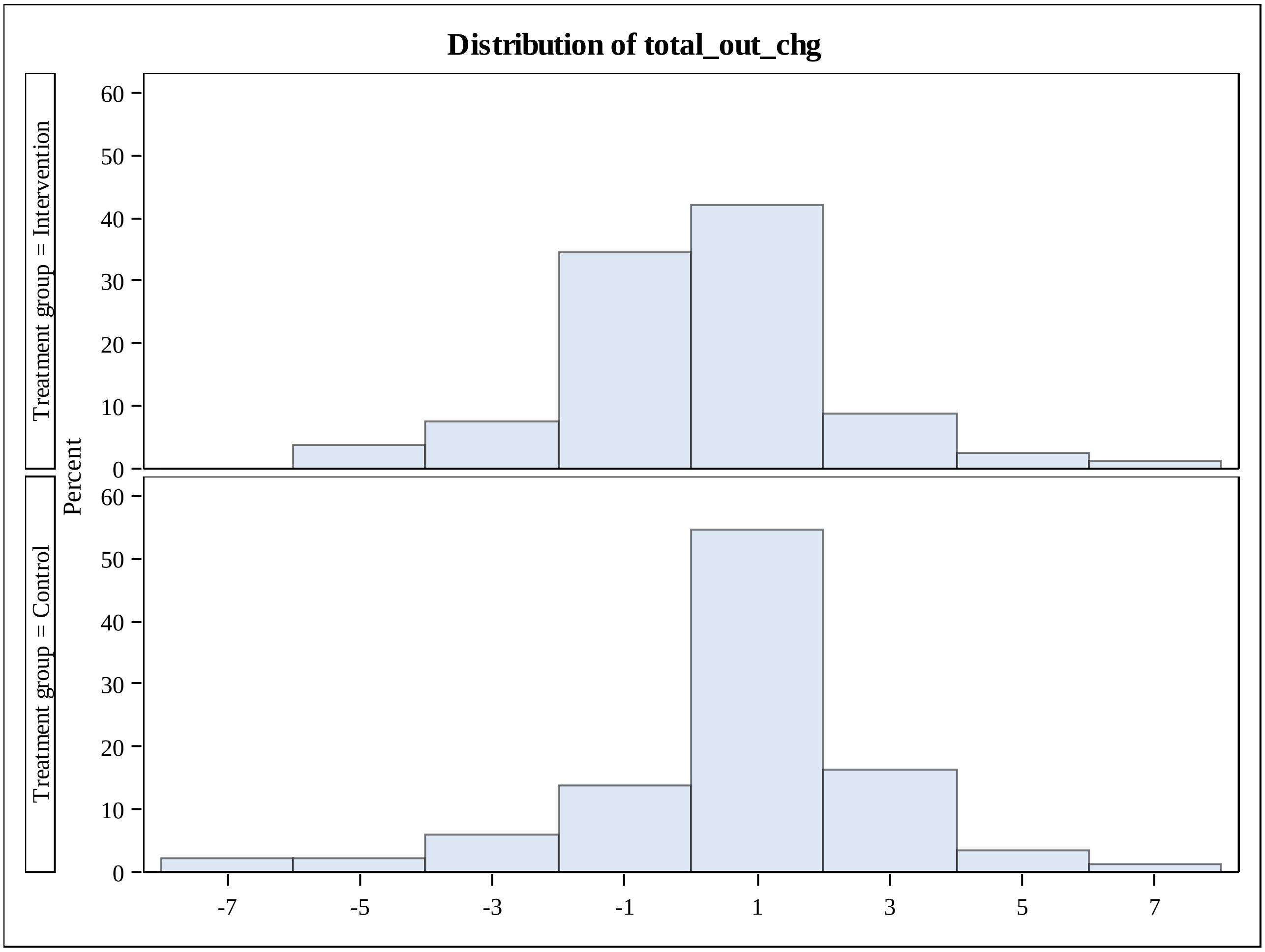

Distribution by treatment group for change from baseline to 12 months
Number of Outpatient Visits

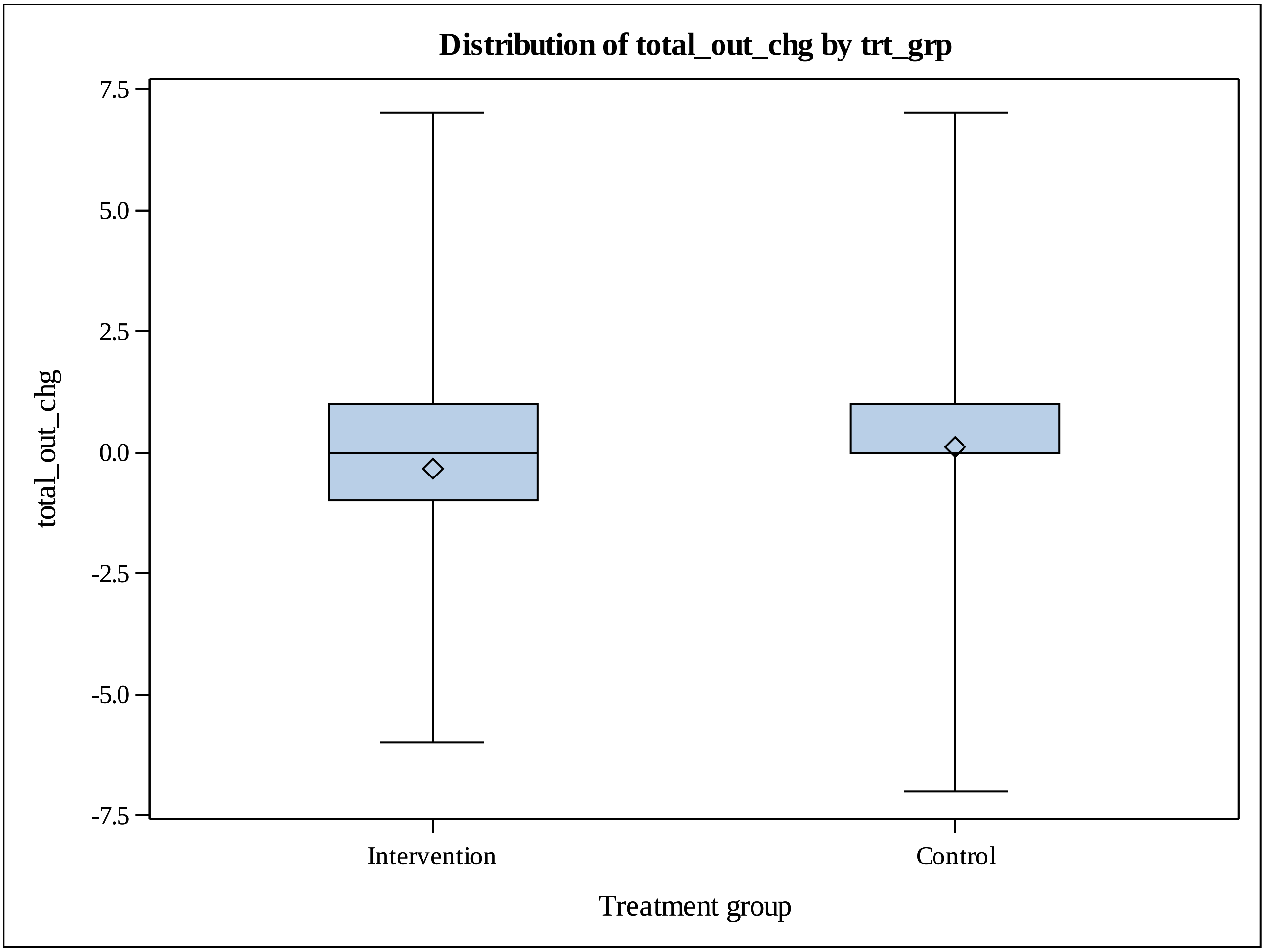

Distribution by treatment group for change from baseline to 12 months
Medication Possession Ratio for All Asthma Medications

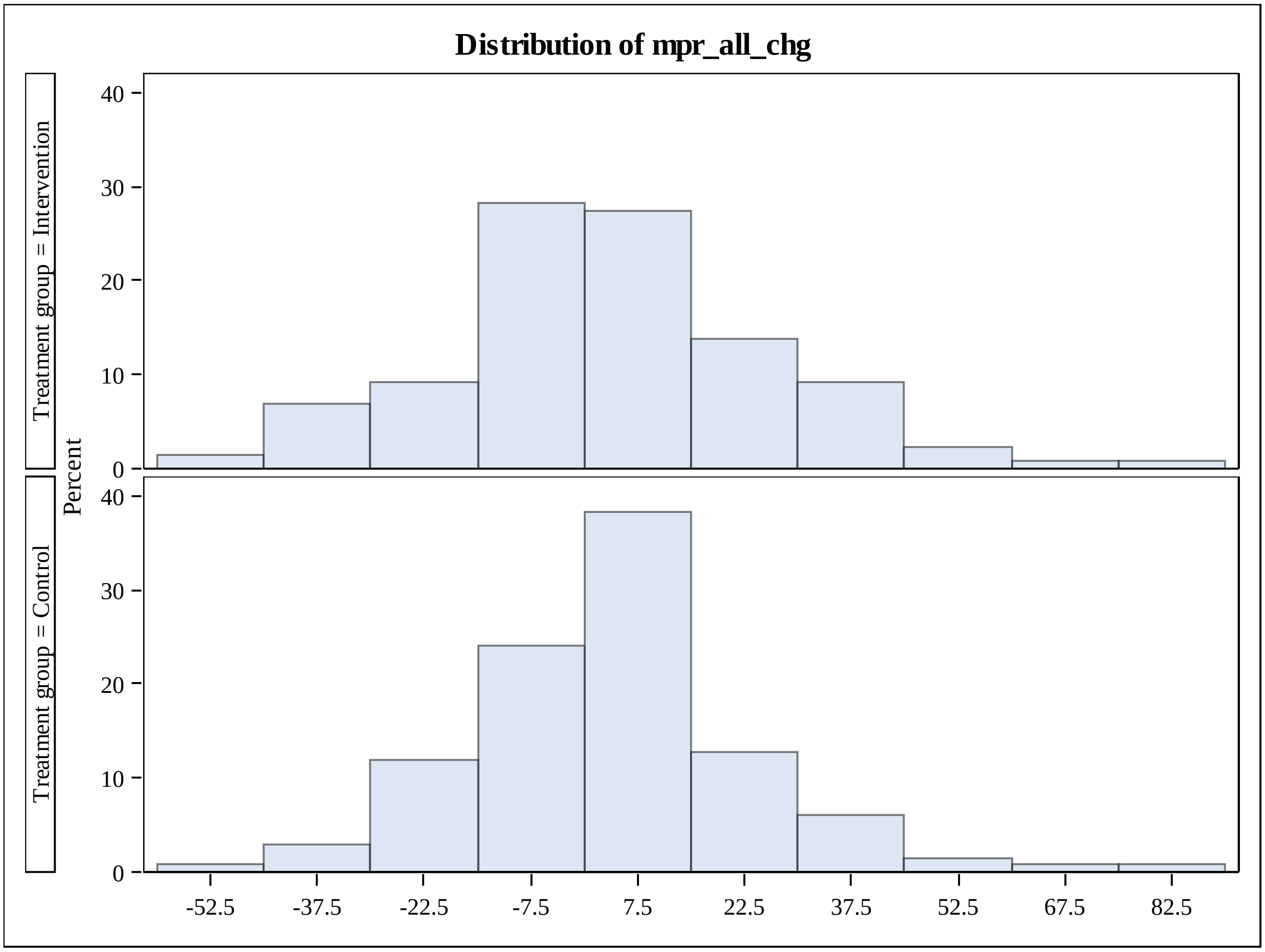

Distribution by treatment group for change from baseline to 12 months
Medication Possession Ratio for All Asthma Medications

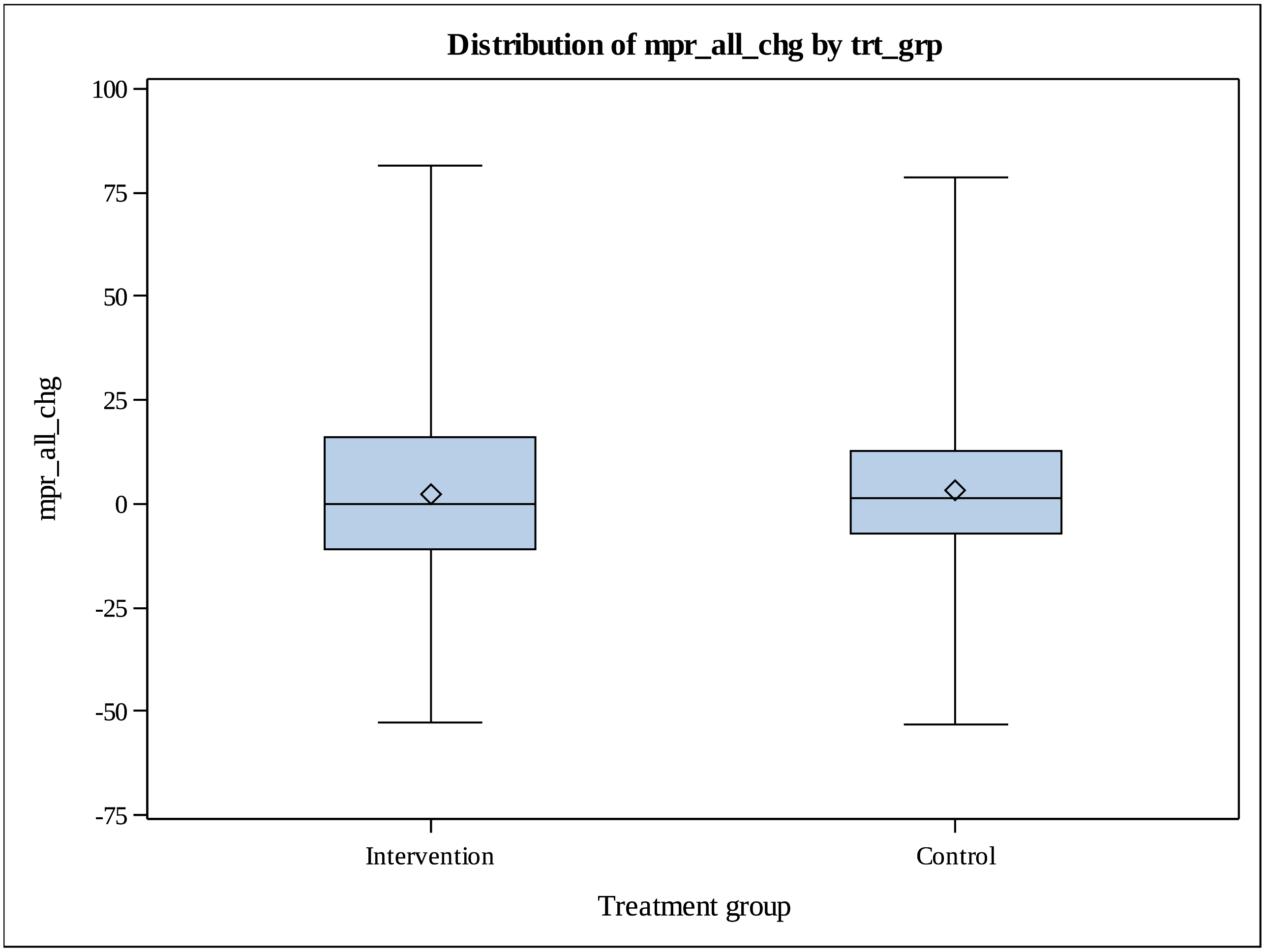

Distribution by treatment group for change from baseline to 12 months Medication Possession Ratio for Asthma Controller Medications

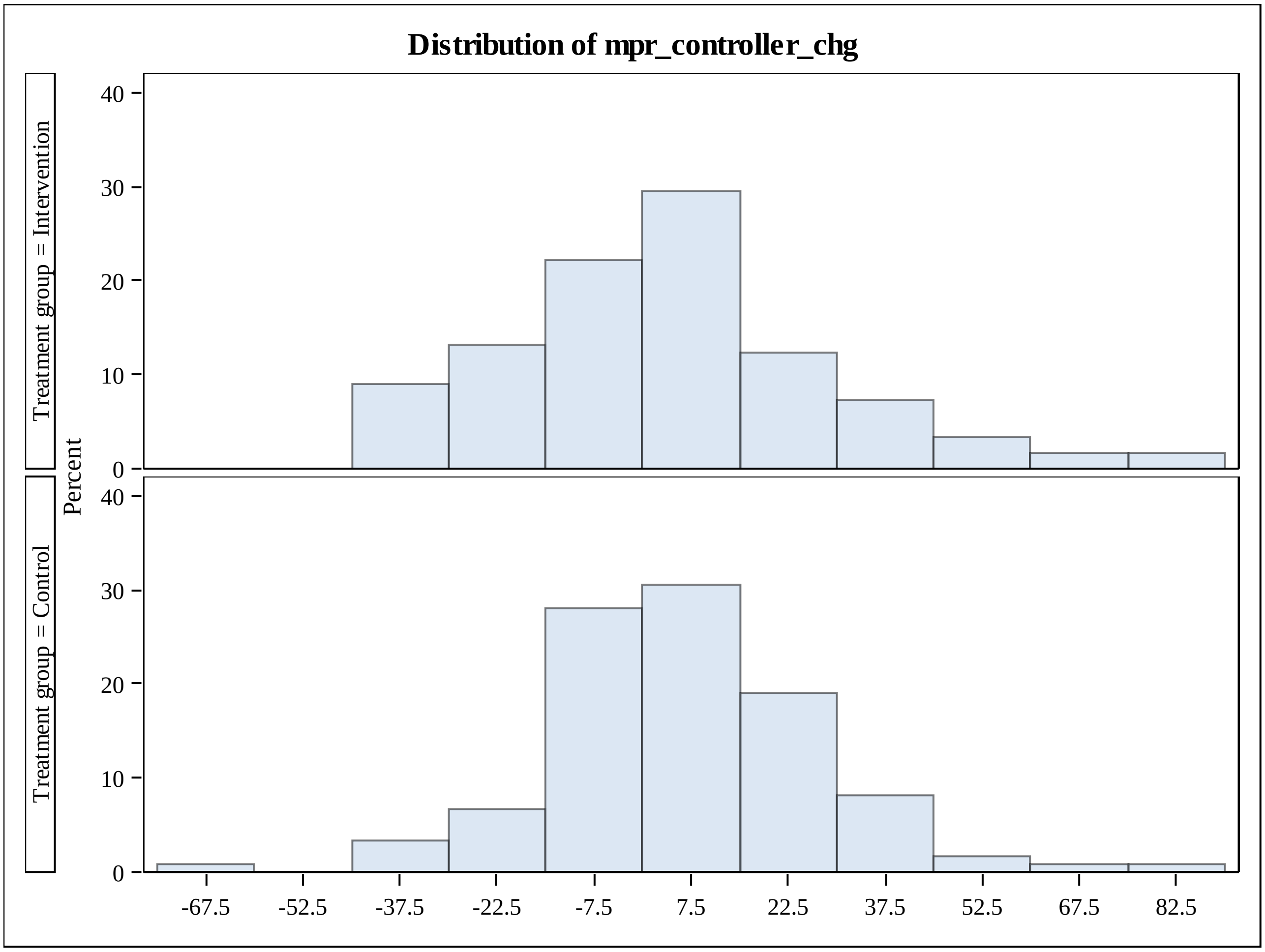

Distribution by treatment group for change from baseline to 12 months
Medication Possession Ratio for Asthma Controller Medications

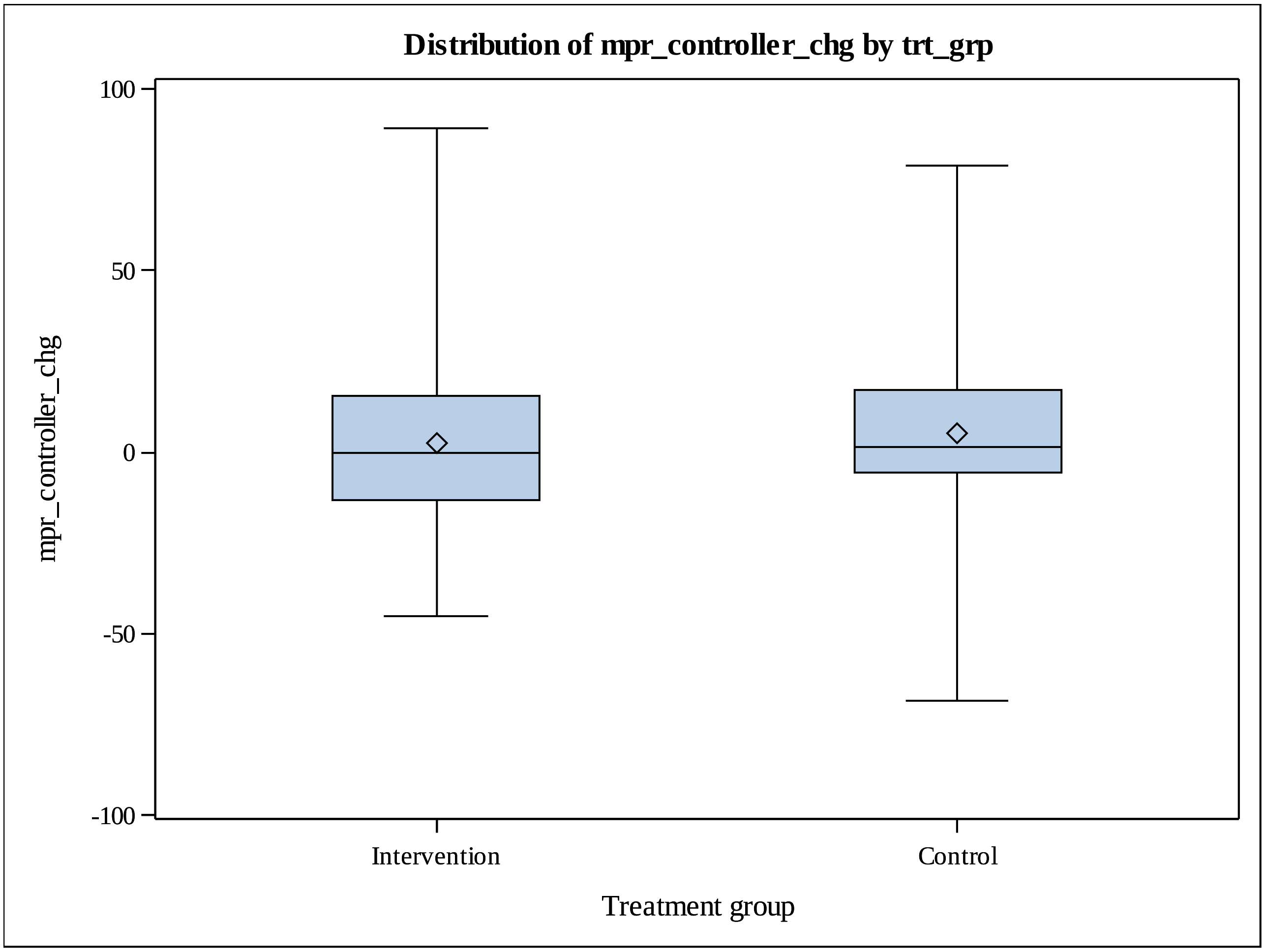

Distribution by treatment group for change from baseline to 12 months
Medication Possession Ratio for Reliever Medications Only

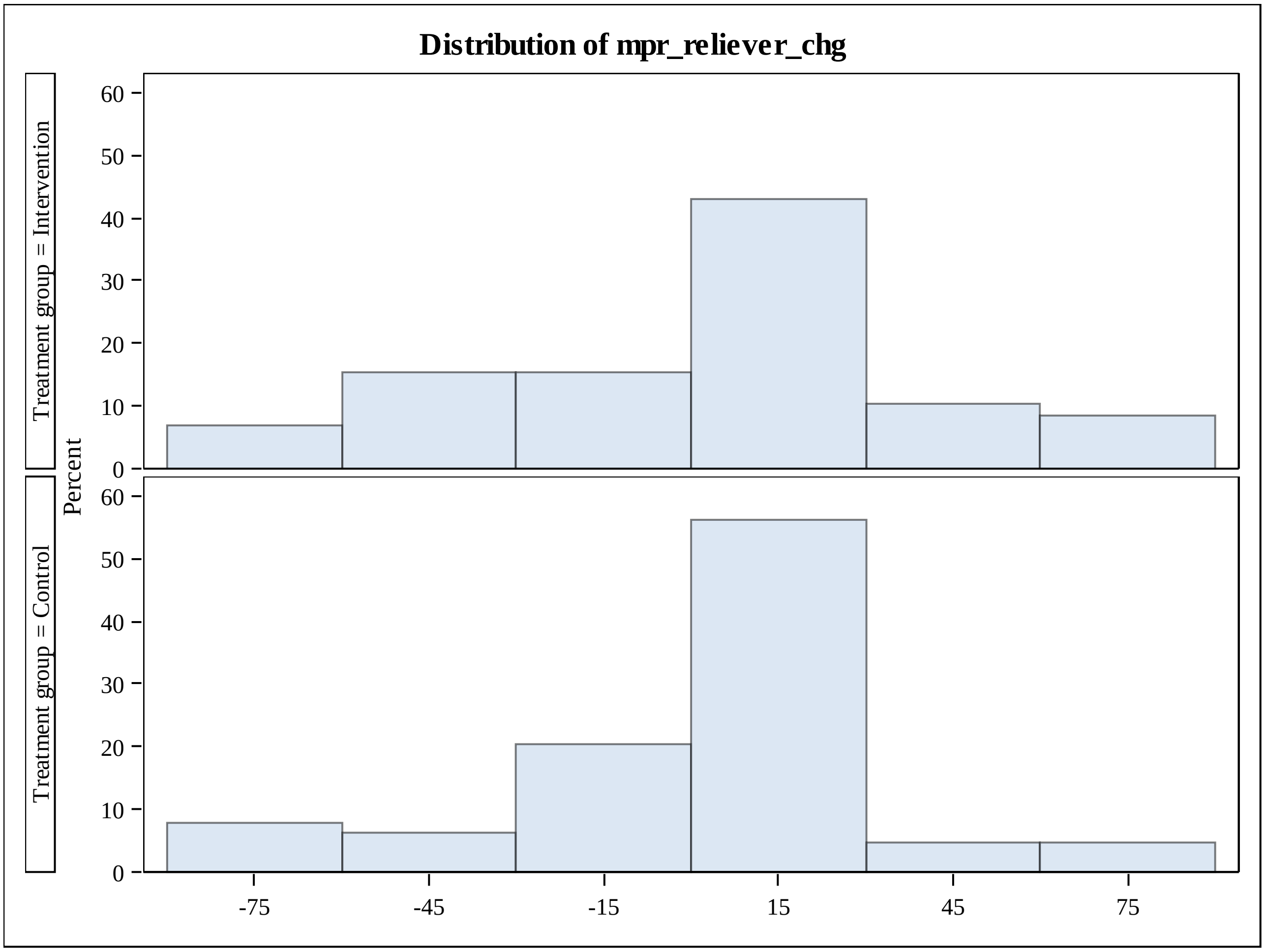

Distribution by treatment group for change from baseline to 12 months
Medication Possession Ratio for Reliever Medications Only

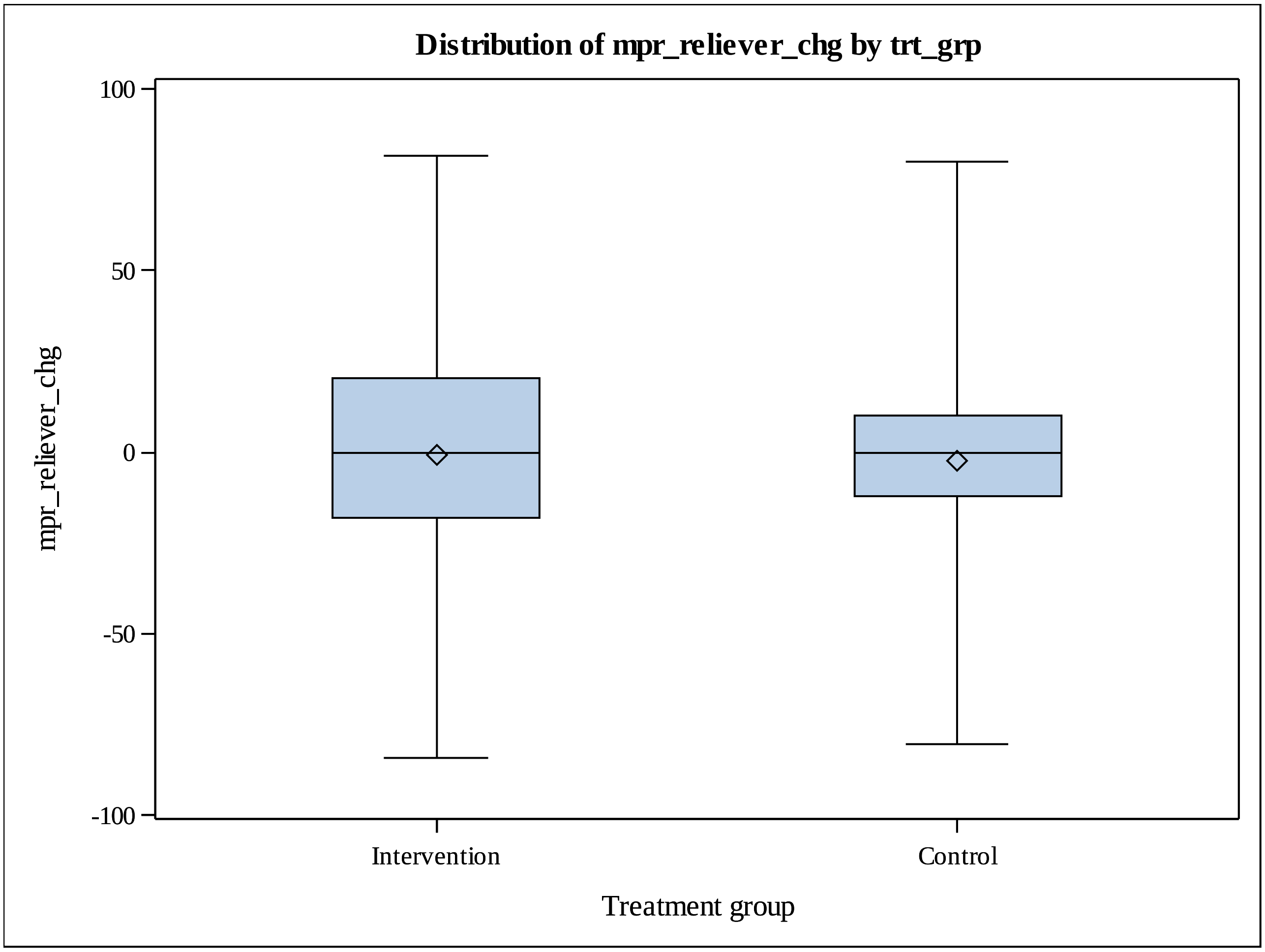

Supplement: Supplementary file 4 [file pone.0170447.s004.doc]
